# RNA m^6^A methylation participates in regulation of postnatal development of the mouse cerebellum

**DOI:** 10.1186/s13059-018-1435-z

**Published:** 2018-05-31

**Authors:** Chunhui Ma, Mengqi Chang, Hongyi Lv, Zhi-Wei Zhang, Weilong Zhang, Xue He, Gaolang Wu, Shunli Zhao, Yao Zhang, Di Wang, Xufei Teng, Chunying Liu, Qing Li, Arne Klungland, Yamei Niu, Shuhui Song, Wei-Min Tong

**Affiliations:** 1 Department of Pathology, Institute of Basic Medical Sciences Chinese Academy of Medical Science, School of Basic Medicine Peking Union Medical College; Neuroscience Center, Chinese Academy of Medical Sciences, Beijing, 100005 China; 2 BIG Data Center, Beijing Institute of Genomics, Chinese Academy of Sciences, Beijing, 100101 China; 3 University of Chinese Academy of Sciences, People’s Republic of China, Beijing, 100049 China; 4 State Key Laboratory of Molecular Oncology, National Cancer Center/Cancer Institute and Hospital, Chinese Academy of Medical Sciences, Peking Union Medical College, Beijing, 100021 China; 5 Department of Microbiology, Oslo University Hospital, Rikshospitalet, Oslo, NO-0027 Norway; 6 Department of Molecular Medicine, Institute of Basic Medical Sciences, University of Oslo, NO-0317 Oslo, Norway

**Keywords:** N^6^-methyladenosine, RNA methylation, ALKBH5, METTL3, Cerebellar development

## Abstract

**Background:**

N^6^-methyladenosine (m^6^A) is an important epitranscriptomic mark with high abundance in the brain. Recently, it has been found to be involved in the regulation of memory formation and mammalian cortical neurogenesis. However, while it is now established that m^6^A methylation occurs in a spatially restricted manner, its functions in specific brain regions still await elucidation.

**Results:**

We identify widespread and dynamic RNA m^6^A methylation in the developing mouse cerebellum and further uncover distinct features of continuous and temporal-specific m^6^A methylation across the four postnatal developmental processes. Temporal-specific m^6^A peaks from P7 to P60 exhibit remarkable changes in their distribution patterns along the mRNA transcripts. We also show spatiotemporal-specific expression of m^6^A writers METTL3, METTL14, and WTAP and erasers ALKBH5 and FTO in the mouse cerebellum. Ectopic expression of METTL3 mediated by lentivirus infection leads to disorganized structure of both Purkinje and glial cells. In addition, under hypobaric hypoxia exposure, *Alkbh5*-deletion causes abnormal cell proliferation and differentiation in the cerebellum through disturbing the balance of RNA m^6^A methylation in different cell fate determination genes. Notably, nuclear export of the hypermethylated RNAs is enhanced in the cerebellum of *Alkbh5*-deficient mice exposed to hypobaric hypoxia.

**Conclusions:**

Together, our findings provide strong evidence that RNA m^6^A methylation is controlled in a precise spatiotemporal manner and participates in the regulation of postnatal development of the mouse cerebellum.

**Electronic supplementary material:**

The online version of this article (10.1186/s13059-018-1435-z) contains supplementary material, which is available to authorized users.

## Background

Epigenetic regulation, including histone modifications, DNA modifications, and non-coding RNAs, plays crucial roles in both embryonic and adult neurogenesis [1]. The most recently discovered regulatory modification, N^6^-methyladenosine (m^6^A), is a highly abundant, so-called epitranscriptomic mark found in mRNAs [2, 3]. Similar to DNA and protein modifications, m^6^A in mRNA is reversible and thus open to dynamic regulation [4, 5]. Levels of RNA methylation are finely balanced through an interplay among m^6^A methyltransferases (writers), demethylases (erasers), and binding proteins (readers) [6]. m^6^A has been shown to regulate RNA processing, including RNA splicing [7, 8], nuclear export [9], RNA degradation [10, 11], and translation [12–14]. In agreement with its functions in RNA metabolism, m^6^A is an important regulatory factor in different cellular processes, including heat shock response [13], DNA damage response [15], cell fate determination [16–19], and innate immunity [20]. In addition to its functions in these cellular processes, in vivo studies in model organisms have revealed crucial roles of m^6^A in spermatogenesis [9, 21–23], embryogenesis [24, 25], and T-cell homeostasis [26].

Although m^6^A is abundant in the brain, its dynamic regulation and biological significance for brain development remain largely unknown [3]. Among the m^6^A-related genes identified, the RNA demethylase FTO is proven to be crucial for various brain functions, including D2R-D3R-GIRK-mediated signaling [27], adult neurogenesis [28], and memory formation [29, 30]. A recent report confirms the role of m^6^A methylation in temporal progression of mammalian cortical neurogenesis and in regulating axon regeneration in the adult mammalian nervous system [31, 32]. To better understand the underlying mechanisms responsible for the temporal and spatial complexity of the brain, it is important to characterize the physiological functions of m^6^A in a context-dependent manner. However, the biological functions of other m^6^A-related genes in different brain regions remain unclear.

The cerebellum consists of a multi-layered structure, rendering it an important model for the study of neuronal cell proliferation, migration, and differentiation [33]. Considerable progress has been made in identifying the epigenetic mechanisms responsible for cerebellar development, such as DNA 5-hydroxymethylation [34] and chromatin accessibility [35]. Interestingly, the cerebellum of *Fto*-deficient mouse was found to be smaller than that of wild-type mouse [28]; however, mechanisms responsible for this growth defect remain unclear. In agreement with the spatial and temporal regulation in the brain, cerebellum-specific methylation has been identified [36]. Despite these findings, the precise role of RNA methylation in the cerebellum and its temporal control during postnatal mouse cerebellar development still await elucidation.

Here we investigated the dynamic regulation of m^6^A in the developing mouse cerebellum and determined its biological significance by disturbing the expression of the m^6^A writer METTL3 or the m^6^A eraser ALKBH5 in mice exposed to hypobaric hypoxia. This study uncovered that the temporal regulation of RNA m^6^A methylation is essential for precise control of postnatal cerebellar development.

## Results

### Temporal regulation of m^6^A methylation in postnatal mouse cerebellum

To understand the biological impact of m^6^A in mouse cerebellum, we first performed m^6^A analysis using postnatal cerebella at day 7 (P7), P14, P21, and P60 (Additional file 1: Table S1). We identified 10,449, 10,266, 10,419, and 10,783 methylated poly(A) RNAs at the four stages, respectively (Additional file 1: Table S2, Additional file 1: Figure S1a–c). Data analysis showed that UGGACU was the most conserved consensus sequence among all m^6^A peaks (Additional file 1: Figure S1d). Distribution analysis of all the m^6^A peaks in mRNAs revealed that their methylation patterns were similar from P7 to P60 (Additional file 1: Figure S1e). Intriguingly, we observed significant enrichment of m^6^A peaks in start codon regions in addition to the coding sequence (CDS) and stop codon regions. A statistical analysis of the m^6^A peaks located in different transcript segments showed that the proportion of m^6^A peaks in start codon regions increased gradually from P7 to P60 (Additional file 1: Figure S1f and Additional file 1: Table S3).

**Fig. 1:**
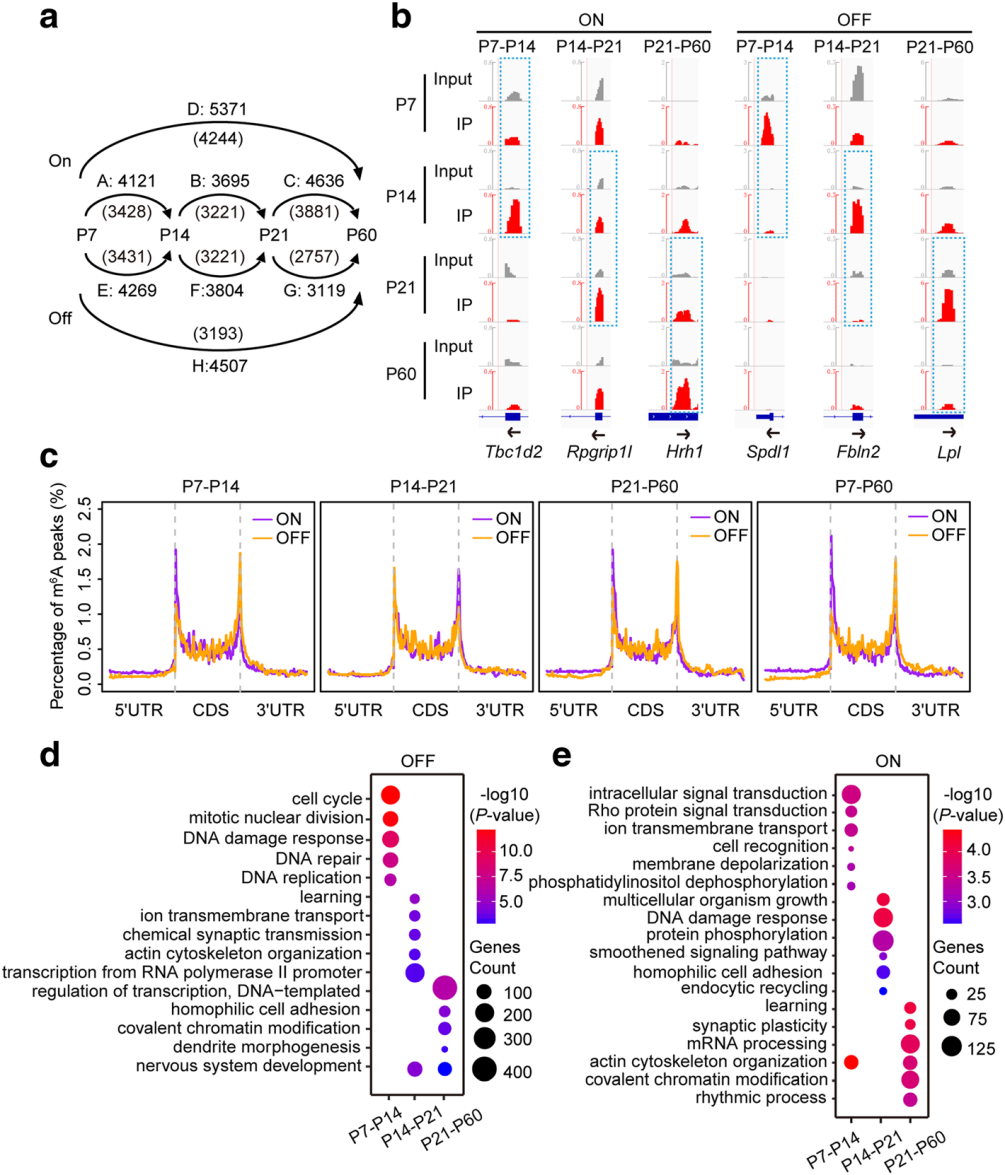
Developmentally regulated RNA methylation during mouse cerebellar development. **a** Numbers of ON or OFF m^6^A switches between P7, P14, P21, and P60 cerebella pairwise comparisons. The numbers of the poly(A) RNAs containing the m^6^A switches are included in parentheses. **b** IGV plots showing examples of ON and OFF m^6^A switches at each transition period. *Grey* reads originate from input libraries and *red* reads originate from m^6^A-IP libraries. *Y-axis* represents normalized numbers of reads count. *Arrows* indicate the direction of gene transcription. *Dashed boxes* indicate the position of ON/OFF switches. **c** Distribution of each type of ON and OFF m^6^A switch along the whole mRNA transcripts. **d**, **e** Most impacted GO biological process terms of the methylated RNAs containing OFF (**d**) or ON (**e**) m^6^A switches across the four developmental stages

To explore how m^6^A changes in postnatal mouse cerebellum, m^6^A peaks were subjected to pairwise comparison between each two adjacent stages. In total, we identified 12,452 “ON” switches (emerging m^6^A peaks in the later stage) and 11,192 “OFF” switches (resolving m^6^A peaks from the former to the later stage) (*P* < 0.05, enrichment score > 1.5) during the postnatal development (Fig. 1a, b and Additional file 1: Figure S1g). The m^6^A ON and OFF switches also exhibited apparent differences in their distribution patterns. The m^6^A ON switches were more likely to be enriched in start codon regions rather than stop codon regions. In contrast, the numbers of m^6^A OFF switches surrounding stop codon regions were higher than that of ON switches (Fig. 1c and Additional file 1: Figure S1h). Together, these data imply that m^6^A marks occurring at earlier or later stages of cerebellar development have different preferences with regard to their distribution along with the whole mRNA transcripts.

To evaluate the biological significance of genes with dynamic RNA m^6^A modification, we next performed Gene Ontology (GO) analysis for those genes with ON/OFF switches (Additional file 2: Table S4). The genes with different types of switches appeared to possess different functions. For example, the genes containing P7–P14 OFF switches were mostly annotated to biological processes such as cell cycle, cell division, and DNA repair (Fig. 1d). In contrast, the newly occurring methylation at P14, P21, and P60 represented by the ON switches was detected in many genes involved in signal transduction, cell adhesion, learning, and synaptic plasticity (Fig. 1e). Together, these data confirm that extensive RNA methylation and demethylation occur over the course of neuronal differentiation in vivo, in both proliferating and fully differentiated neural cells. Moreover, the distinct functions of genes containing m^6^A OFF or ON switches suggest that m^6^A is a prerequisite for those genes to exert their functions at each developmental stage.

### Temporal-specific m^6^A methylation acts in concert with cerebellar developmental control

**Fig. 2:**
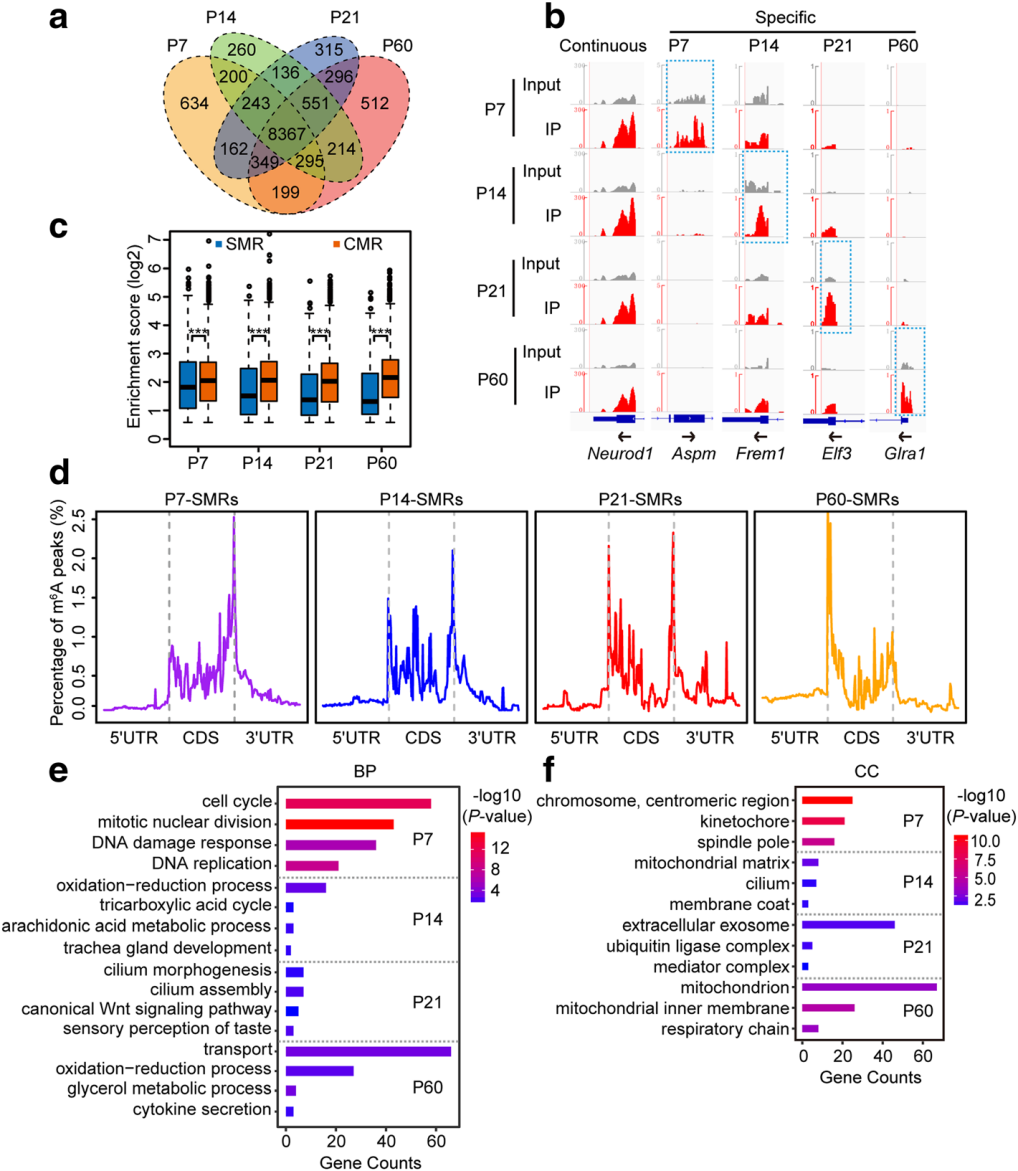
Distinct features of temporal-specific m^6^A methylation during postnatal development of the mouse cerebellum. **a** Venn diagram showing the numbers and relationship of methylated poly(A) RNAs in the mouse cerebellum from P7 to P60. **b** IGV plots showing examples of continuous and temporally specific methylation. *Grey* reads originate from input libraries and *red* reads originate from m^6^A-IP libraries. *Y-axis* represents normalized numbers of read counts. *Arrows* indicate the direction of gene transcription. *Dashed boxes* indicate the position of temporal-specific m^6^A peaks. **c** Box plots showing the relative methylation levels of SMRs and CMRs as evaluated by the enrichment scores of m^6^A peaks. *SMR* specifically methylated RNA, *CMR* continuously methylated RNA. ***P < 0.001 by Wilcoxon test. **d** Normalized distribution of temporal-specific m^6^A peaks in each developmental stage along the whole mRNA transcripts. **e**, **f** Most impacted GO biological process terms (*BP*) (**e**) and cellular component terms (*CC*) (**f**) of SMRs in the four developmental stages

As m^6^A is developmentally regulated in mouse cerebella, we next analyzed the methylation profiles over the four postnatal stages. We found 8367 continuously methylated RNAs (CMRs) throughout cerebellar development, together with 634, 260, 315, and 512 specifically methylated RNAs (SMRs) at P7, P14, P21, and P60, respectively (Fig. 2a, b and Additional file 1: Figure S2a). We then investigated whether the SMRs and CMRs possessed different features in their methylation during postnatal cerebellar development. Compared with the SMRs, the CMRs exhibited higher levels of both methylation and expression throughout the developmental process. Moreover, the methylation levels of SMRs displayed a gradual reduction from P7 to P60, while their expression levels changed in the opposite direction (Fig. 2c and Additional file 1: Figure S2b–c). Given the different distribution between ON and OFF switches (Fig. 1c and Additional file 1: Figure S1h), we further analyzed the distribution of m^6^A peaks in continuously and temporal-specifically methylated mRNAs. In the mouse cerebellum of P7, the temporal-specific m^6^A peaks were significantly enriched at stop codon regions, while the P60-specific m^6^A peaks were mostly enriched in start codon regions. The P14- or P21-specific m^6^A peaks displayed an apparent transition from stop codon to start codon during progression of cerebellar development (Fig. 2d). Furthermore, we quantified the numbers of temporal-specific m^6^A peaks located in the five regions of mRNA transcripts. From P7 to P60, we observed a sharp increase in the numbers of temporal-specific m^6^A peaks in start codon regions, as well as a decrease in the CDS and stop codon regions (Additional file 1: Figure S2d). To ensure that these changes were real rather than artifacts, we randomly extracted the same numbers of CMR peaks as those of temporal-specific peaks and repeated the distribution analysis. Unlike the temporal-specific peaks shown in Fig. 2d, these CMR peaks displayed similar distribution patterns from P7 to P60 (Additional file 1: Figure S2e). The majority of cells in the cerebellum of P7 are proliferating and immature, while the cells in the cerebellum at P60 are fully differentiated. To confirm that such a dynamic distribution pattern was related to the cell states, we compared the RNA methylation profiles in mouse cerebellum at P7 and P60 directly using the peaks identified by exomePeak. Although the proportion of m^6^A peaks located in CDS were similar between the two stages, we observed a higher percentage of P7-specific m^6^A peaks near stop codon regions, but a higher percentage of P60-specific m^6^A peaks in start codon regions (Additional file 1: Figure S2f). These data show that for those temporal-specifically methylated RNAs, the patterns of m^6^A deposition onto RNA exhibit significant changes along with the progression of cerebellar development.

To explore the functional significance of temporal-specific methylation, we next performed GO analysis for those genes with continuous or temporal-specific methylation. In line with the progression of cerebellar development, the four groups of genes containing temporal-specific m^6^A marks were annotated to different biological processes (Fig. 2e and Additional file 3: Table S5). Genes with P7-specific methylation (such as *Aspm* in Fig. 2b) were enriched in processes such as cell cycle, cell division, and DNA damage response, which are prerequisites in proliferating neural cells. In contrast, many genes displaying P60-specific methylation (such as *Glra1* in Fig. 2b) were annotated to biological processes including transport, oxidation-reduction process, and metabolic processes, which are required for mature neuronal activities. In agreement with their diverging biological functions, the genes with temporal-specific methylation were also characteristic of their distinct cellular localization (Fig. 2f). In contrast, genes encoded by the P7 and P60 CMRs exhibited high similarity in their annotation of enriched biological processes and cellular components (Additional file 1: Figure S2g and Additional file 3: Table S5). Together, these results suggest that the majority of genes with continuous methylation play a fundamental role throughout cerebellar development, while temporal-specific methylation only occurs at specified times for those genes, thus ensuring the proper progression of cerebellar development in a temporal-dependent manner.

In parallel to the methylation analysis using the m^6^A peaks identified using exomePeak, we re-analyzed the m^6^A-seq data at P7 and P60 using another canonical peak-calling algorithm, MACS2. The P7 SMRs, P60 SMRs, and CMRs exhibited distinct features in their relative methylation levels, distribution patterns of the m^6^A peaks, and their functional annotations (Additional file 1: Figure S3 and Additional file 4: Table S6). The very high similar results from the two sets of independent analysis further strengthened our awareness of the importance of temporal specific m^6^A methylation during mouse cerebellar development. We then evaluated how the RNA methylation impacted on gene expression. When each sample was analyzed individually, RNA methylation and expression levels at the global scale were negatively correlated with each other (Additional file 1: Figure S4a). Next, pairwise comparison of RNA expression between each two adjacent stages identified a total of 2451 (*P* value < 0.05) differentially expressed RNAs throughout development (Additional file 1: Figure S4b), among which approximately 90% were validated by another parallel differential expression analysis using a STAR/edgeR package. Cluster analysis of all expressed RNAs from P7 to P60 produced six clusters of gene expression patterns (Additional file 1: Figure S4c), among which 839 RNAs showed either positive (674) or negative (165) correlation between methylation and expression levels across the four stages (Additional file 5: Table S7). Results of the cluster and subsequent GO analyses suggest that for these RNAs, m^6^A participates in the developmental control of the mouse cerebellum through modulating their gene expression (Additional file 1: Figure S4d, e).

### Altered expression of either METTL3 or ALKBH5 causes defective mouse cerebellar development

**Fig. 3:**
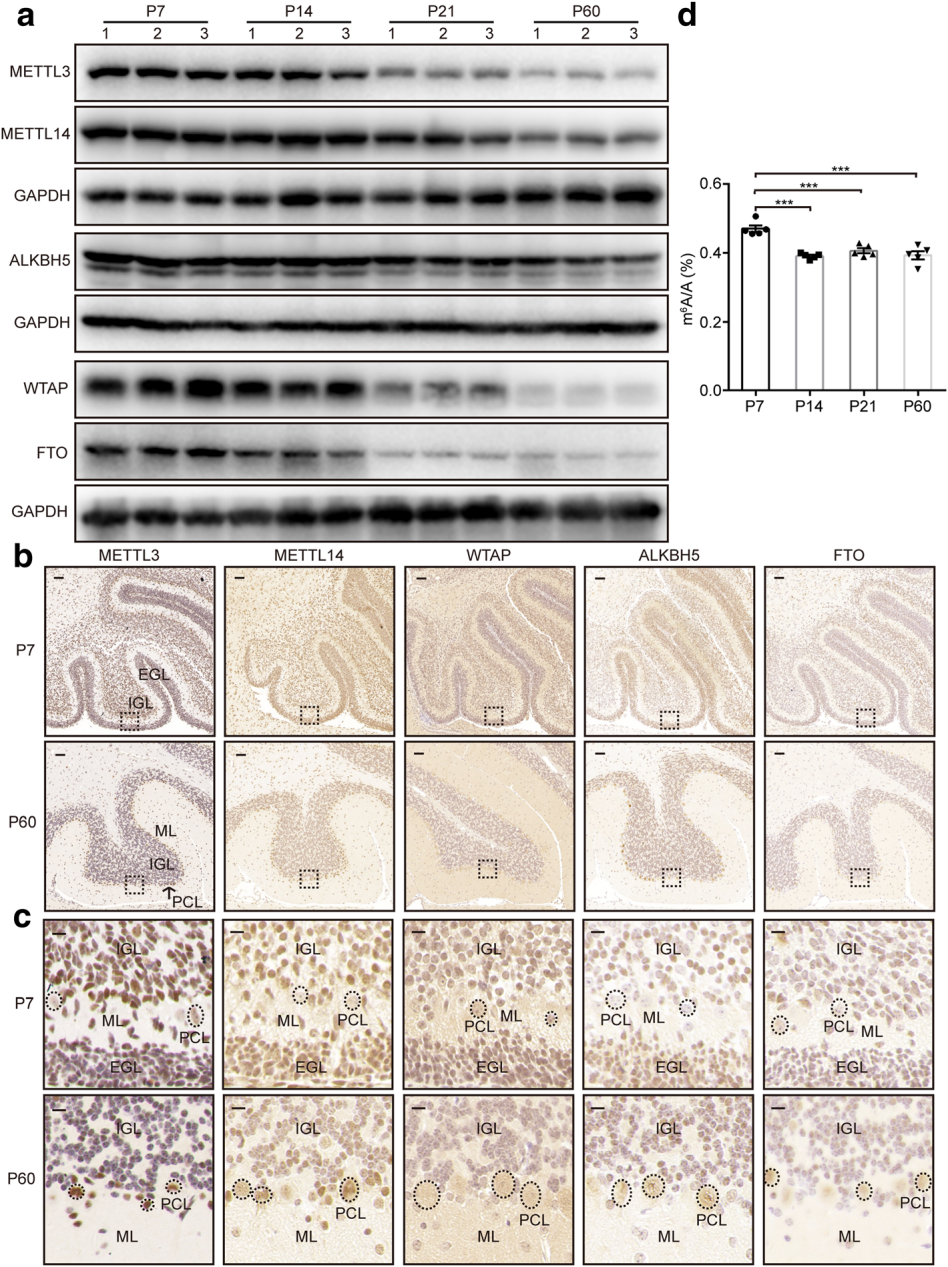
Dynamic expression of m^6^A-related genes and RNA methylation levels during mouse cerebellar development. **a** Western blot analysis showing the expression levels of m^6^A methyltransferases and demethylases in the mouse cerebellum from P7 to P60. *n* = 12 biological replicates. GAPDH is used as an internal control. **b**, **c** Immunostaining of mouse cerebellum at P7 and P60 using antibodies against the five m^6^A-related proteins. *n* = 4 biological replicates. Enlarged images in the *dashed boxes* are shown in **c**. *Scale bar* in **b** represents 50 μm, and *scale bar* in **c** represents 10 μm. *EGL* external granule cell layer, *IGL* internal granule cell layer, *PCL* Purkinje cell layer, *ML* molecular layer. The *dashed circles* indicate the representative Purkinje cells. **d** Dynamic poly(A) RNA m^6^A methylation levels during mouse cerebellar development. Mouse cerebella at P7, P14, P21, and P60 were included in this assay. Experiments were repeated three times using 15 mice in total. Representative data are shown here. ***P < 0.001

To gain insights into how the dynamic m^6^A methyl marks are regulated during cerebellar development, we analyzed the protein expression profiles of three m^6^A writers (METTL3, METTL14, and WTAP) and two m^6^A erasers (ALKBH5 and FTO). All five proteins were highly expressed at the early stage of cerebellar development (P7) and showed a gradual reduction towards the maturation of cerebellar neurons (P60) (Fig. 3a). Given the spatiotemporal specificity of gene expression in the brain, we performed immunohistochemical staining to detect their expression in situ. In the cerebellum at P7, all five proteins were detected in the external granule cell layer (EGL), Purkinje cell layer (PCL), and inner granule cell layer (IGL); however, their expression levels varied among different types of cells (Fig. 3b). Furthermore, along with the progression of cerebellar development, we observed a reduction of their expression levels in internal granular layers but an elevation in Purkinje cells (Fig. 3c).

**Fig. 4:**
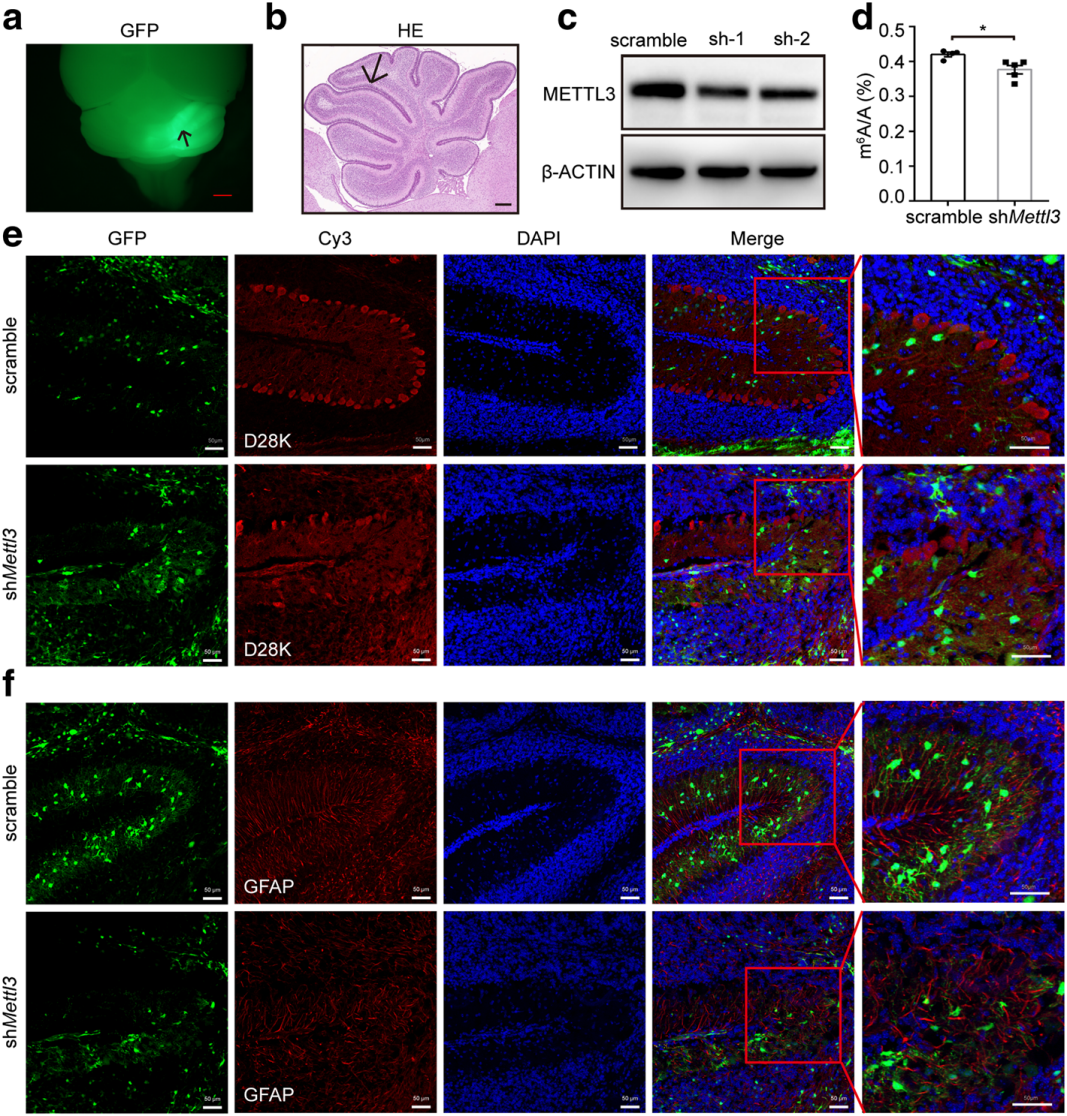
Altered morphology in the mouse cerebellum infected with lentivirus for *Mettl3* knockdown. **a, b** GFP fluorescence image (**a**) and hematoxylin-eosin (*HE*) staining (**b**) of the mouse cerebellum dissected 10-days post-lentivirus infection. *Arrows* indicate the position of injection. *Scale bar* in **a** represents 1 mm. *Scale bar* in **b** represents 200 μm. **c** Western blot analysis to confirm the knockdown efficiency of *Mettl3* in the neuro2a cell line. β-ACTIN was used as an internal control. sh-1 and sh-2 represent two kinds of *Mettl3* knockdown lentiviral vectors. sh-1 was used for subsequent in vivo analysis. **d** Decreased m^6^A levels resulting from *Mettl3* knocking down as analyzed using UHPLC-MS/MS. *n* = 4. *P < 0.05. **e**, **f** Immunofluorescence staining with antibodies against Calbindin-D28K (**e**) and GFAP (**f**) was performed to detect Purkinje cells (**e**) and astrocytes (**f**) upon lentivirus infection (*n* = 3 biological replicates). GFP fluorescence indicates the area with *Mettl3* knockdown. Sections were counterstained with DAPI to visualize the nuclei. *Scale bar* represents 100 μm

In agreement with the dynamic expression of the m^6^A-related proteins, the global m^6^A levels of poly(A) RNA also decreased from P7 to P60 (Fig. 3d). The highest m^6^A level at P7 suggests that m^6^A methylation plays an important role at the earlier developmental stage. To confirm this, we reduced local m^6^A levels in the developing cerebellum (P7) by knocking down *Mettl3* using lentivirus system and examined the consequent morphological changes (Fig. 4a–d). Purkinje cells in the control group displayed an orderly alignment along the outer surface of IGLs with dendritic arborization. However, knockdown of *Mettl3* resulted in a severe alteration in Purkinje cell numbers and laminal structure and and stunted dendrites (Fig. 4e). Moreover, GFAP immunostaining revealed that glial cell fibers were severely disorganized in *Mettl3*-knocked down regions (Fig. 4f). We also examined the effect of *Mettl3* overexpression on cerebellar development (Additional file 1: Figure S5a, b). Although the global m^6^A levels increased modestly (Additional file 1: Figure S5c), overexpression of *Mettl3* led to apparent morphology changes, as was observed in the *Mettl3*-knocked down cerebellum (Additional file 1: Figure S5d-S5e), suggesting that appropriate m^6^A levels are crucial for cerebellar development.

**Fig. 5:**
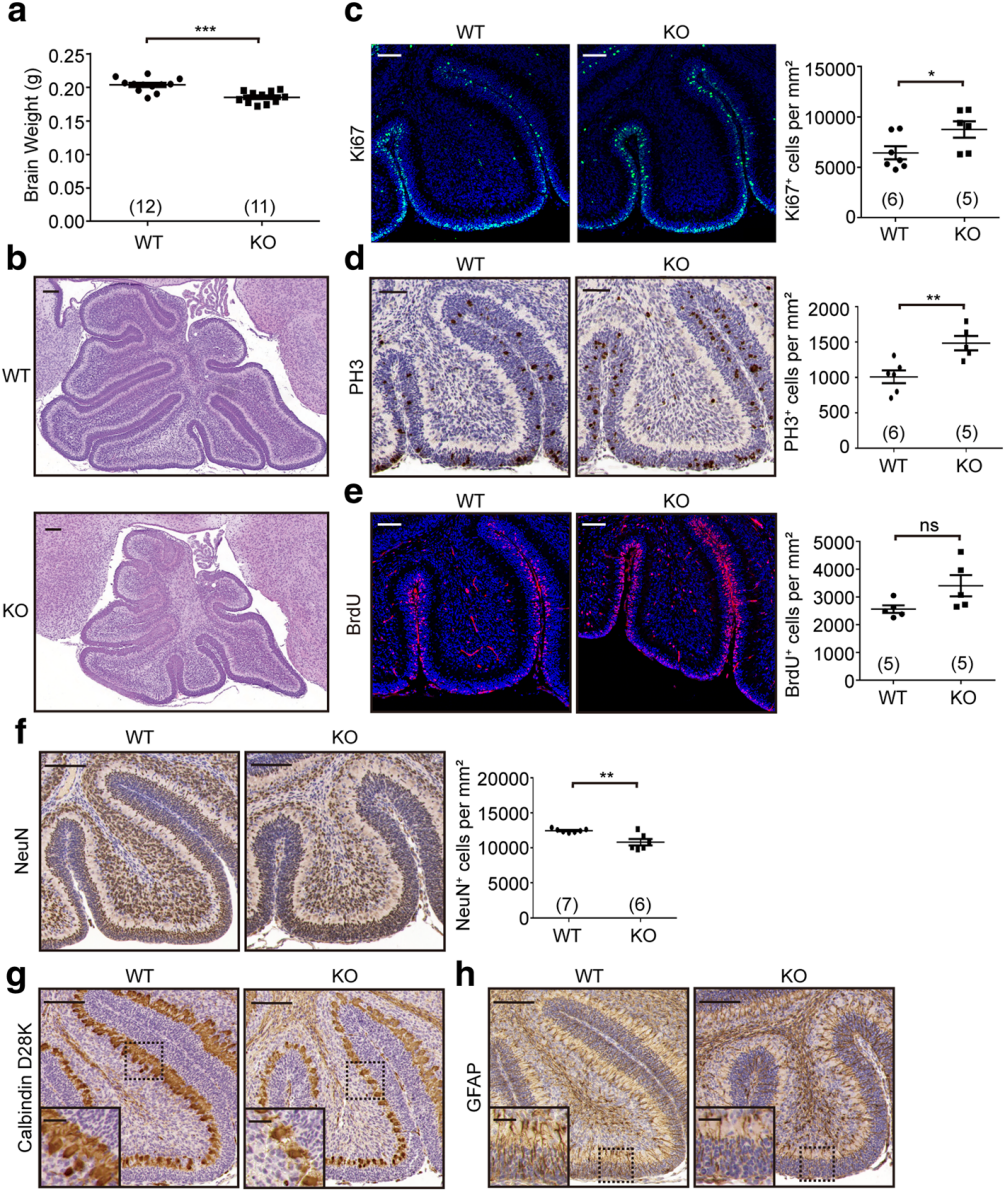
Defective cerebellar development in *Alkbh5* knockout mice after being exposed to hypobaric hypoxia for 48 h. **a** Brain weight of individual wild-type (*WT*) and *Alkbh5* knockout (*KO*) mice. Numbers of biological replicates are included in parentheses. *Black lines* in the graph indicate the mean. ***P < 0.001. **b** Representative HE staining images of wild-type (*WT*) and *Alkbh5* knockout (*KO*) mouse cerebellum (P7). *n* = 7 for WT and KO, respectively. *Scale bar*, 100 μm. **c–f** Representative images from immunostaining analysis with antibodies against Ki67 (**c**), phospho-H3 (PH3) (**d**), BrdU (**e**), and NeuN (**f**) in the cerebellum of WT and KO mice. Quantification of immuno-reactive cells is shown in the *right panels*. Numbers of biological replicates are included in parentheses. *Black lines* in the graph indicate the mean. *P < 0.05, **P < 0.01, *ns* not significant. Sections in **c** and **e** were stained with DAPI and sections in **d** and **f** were counterstained with eosin to visualize nuclei. *Scale bar*, 100 μm. **g**, **h** Representative images from immunohistochemical analysis with antibodies against Calbindin-D28K (**g**) and GFAP (**h**) to detect Purkinje cells (**g**) and astrocytes (**h**). *n* = 7 for WT and KO, respectively. *Scale bar*, 100 μm. *P < 0.05, **P < 0.01, ***P < 0.001, *ns* not significant

Given the spatiotemporal expression of ALKBH5 in the mouse cerebellum, we next tested its role in cerebellar development. Compared to that of wild-type (WT) mice, the cerebellum of *Alkbh5*-knockout (KO) mice lacked any detectable changes in weight and morphology (Additional file 1: Figure S6). Considering the potential functions of FTO and other undefined demethylases in the cerebellum, we speculated that this phenomenon was likely due to genetic redundancy and a compensatory response [37]. To overcome these compensatory mechanisms, we made use of the fact that the mammalian brain is sensitive to neuronal damage by hypoxia [38], while ALKBH5 is unique among the ALKB families in its response to hypoxia stimulation [39]. Therefore, we challenged the mice to extreme physiological conditions in order to exhaust the potential compensatory mechanisms. We propose that ALKBH5 might be involved in the protective mechanism of the brain against damage caused by hypoxia. To confirm this, WT and KO mice were exposed to hypobaric hypoxia for 48 h. We found that in KO mice the sizes of the whole brain and the cerebellum were significantly reduced compared to their littermate controls (Fig. 5a, b). Immunostaining analysis revealed a significant increase in the numbers of Ki67^+^ proliferating cells and phospho-histone 3 (PH3^+^) mitotic cells in the EGL compared to the WT counterparts (Fig. 5c, d). Consistently, we also detected an increase in the number of cells in the S phase of the cell cycle (positive BrdU immunoactivity) in the cerebellum of KO mice (Fig. 5e). Furthermore, the number of mature neurons in the IGL of *Alkbh5*-deficient cerebellum was significantly reduced as reflected by NeuN immunostaining (Fig. 5f). These data suggest that *Alkbh5* deficiency disrupted the normal progression of granular neurons from proliferation to differentiation. In addition, we found that *Alkbh5* deficiency considerably reduced dendritic arborization of Purkinje cells (Fig. 5g), concomitant with an increase in disorganization of the radial fibers in glial cells (Fig. 5h). Following hypoxic treatment and return to normoxic conditions, we examined the cerebellar sections again at another stage (P14). The internal granule neurons, Purkinje cells, and glial cells exhibited no visible difference between WT and KO mice (Additional file 1: Figure S7a–c). However, we could still observe a slight increase in the numbers of proliferating granule cells as indicated by Ki67 and PH3 immunoreactivity (Additional file 1: Figure S7d, e). In addition, the cerebellum in *Alkbh5* KO mice remained smaller than that of the littermate controls (Additional file 1: Figure S7f). These data indicate that in spite of the undefined compensatory response to partially recover the morphology changes, *Alkbh5* deficiency had a profound and deleterious effect on cerebellar development under hypoxic conditions.

Together, the abnormal cerebellar development resulting from either ectopic expression of *Mettl3* or *Alkbh5* deficiency demonstrates that proper orchestration of the spatiotemporal expression of m^6^A writers and erasers is necessary for mouse cerebellar development.

### Imbalance of RNA m^6^A methylation in the *Alkbh5*-deficient mouse cerebellum exposed to hypobaric hypoxia

**Fig. 6:**
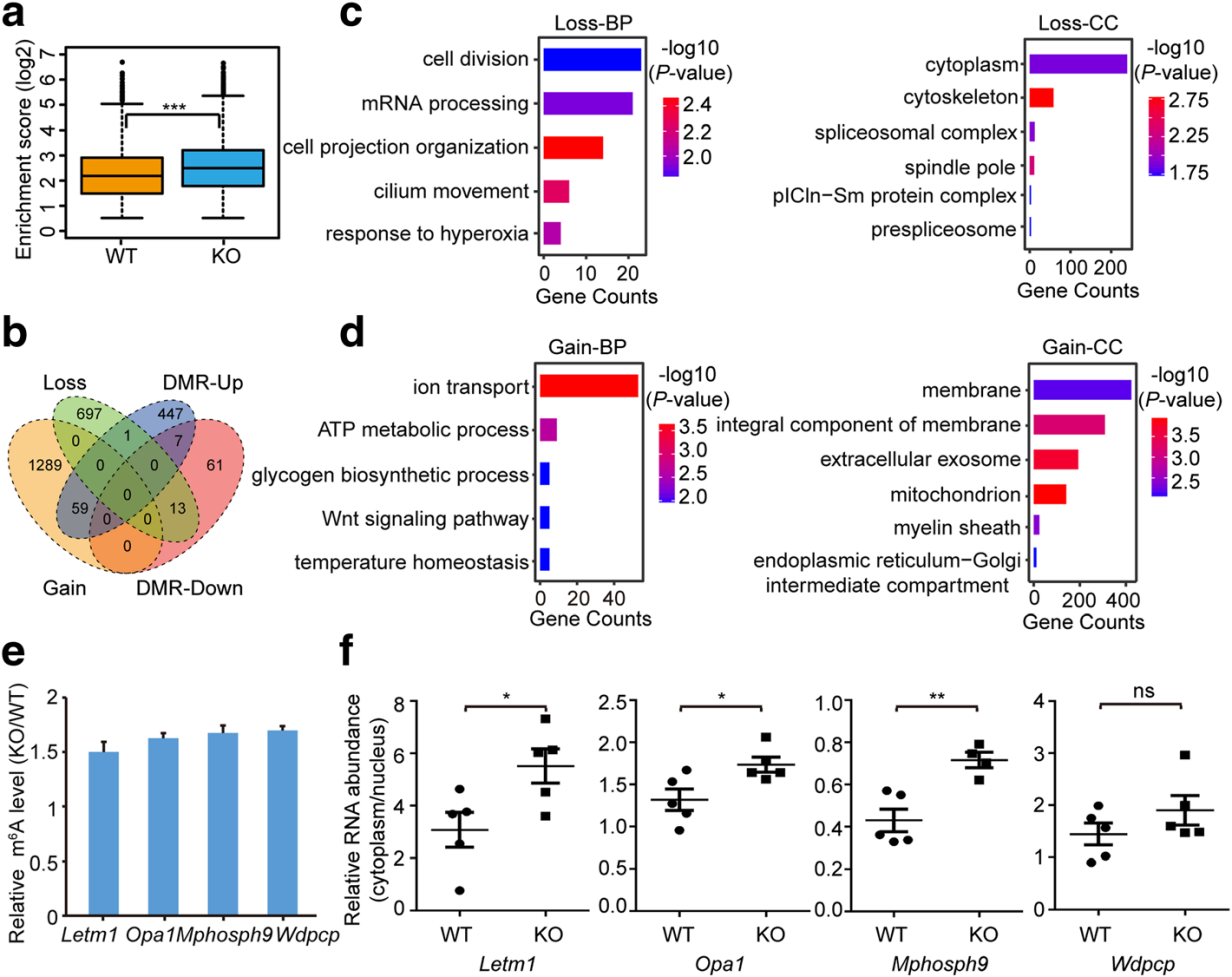
Imbalanced m^6^A RNA methylation in the cerebellum of *Alkbh5*-deficient mouse exposed to hypobaric hypoxia. **a** Boxplots showing the relative methylation levels of all m^6^A peaks between wild-type (*WT*) and *Alkbh5*-deficient (*KO*) mouse cerebellum. ***P < 0.001 by Wilcoxon test. **b** Venn diagram showing the relationship among the RNAs containing gain-of-methylation (*Gain*), lost-of-methylation (*Loss*), and differentially methylated RNAs (*DMRs*). **c**, **d** GO analysis of genes encoded by RNAs containing loss- (**c**) or gain-of**-**methylation (**d**) in KO mouse cerebellum. *BP* biological process, *CC* cellular component. **e** Gene-specific m^6^A-IP-qPCR results showing the relative methylation levels of four RNAs in the cerebellum of KO mice compared to those in WT mice. *n* = 7. **f** Subcellular localization of the four RNAs as shown by the ratio of RNA abundance between the cytoplasmic and nuclear fractions isolated from the cerebellum of WT and KO mice. *n* = 10 for WT and 8 or 10 for KO, respectively. *P < 0.05, **P < 0.01, *ns* not significant

To examine how *Alkbh5* deficiency affects m^6^A methylation of the cerebellar poly(A) RNA, we first performed UHPLC-MS/MS analysis on the poly(A) RNA from cerebella at P7 and found a slight increase in the global m^6^A levels in the KO mice exposed to hypobaric hypoxia (Additional file 1: Figure S8a). To gain more detailed insight into the epitranscriptome-wide changes of all methylated RNAs, we performed m^6^A-seq using the poly(A) RNAs from both WT and KO mice exposed to hypobaric hypoxia (Additional file 1: Table S1). A slight increase in the number of m^6^A peaks and methylated RNAs was observed in the cerebella of KO mice (Additional file 1: Table S2), as well as higher enrichment scores of m^6^A peaks (Fig. 6a). In addition, we identified 1348 poly(A) RNAs with gain of methylation and 711 poly(A) RNAs with loss of methylation in KO mice cerebellum (P7) (Fig. 6b; Additional file 1: Figure S8b, c). By comparing those abnormally methylated RNAs to the SMRs during cerebellar development of the WT mice, we found that upon *Alkbh5* deficiency, 53 original P7 SMRs lost their methylation, while 42 original P14 SMRs, 53 P21 SMRs, and 104 P60 SMRs became methylated at P7. In addition to the gain- and loss-of-methylation RNAs, we also identified a considerable number of RNAs exhibiting a significant increase (514) or decrease (81) in their methylation levels (Fig. 6b). Therefore, in spite of a mild increase of m^6^A at the global level, *Alkbh5* deficiency led to disordered m^6^A levels of thousands of RNAs.

We next performed GO analysis to evaluate whether the defective cerebellar development in KO mice resulted from imbalanced m^6^A methylation (Fig. 6c, d and Additional file 1: Figure S8e). Partial or complete loss of methylation mainly occurred to the genes participating in the control of cell division (such as *Cenpe* in Additional file 1: Figure S8c), cell cycle (such as *Cdca2* in Additional file 1: Figure S8d), and cell projection organization (such as *Erbb4* in Additional file 1: Figure S8d). In contrast, the hypermethylated RNAs were found to be related to metabolic processes (such as *Ccr5* in Additional file 1: Figure S8c), ion transport (such as *Camk2g* in Additional file 1: Figure S8d), and axon guidance (such as *Rora* in Additional file 1: Figure S8d).

Previous studies showed that ALKBH5 regulates RNA nuclear export via an m^6^A-dependent manner. We therefore asked whether the changes in m^6^A methylation resulting from *Alkbh5* deficiency also affect the RNA nuclear export in vivo. Based on functional enrichment analysis, we chose several genes in different functional pathways, such as cell division (*Mphosph9*) [40], membrane potential (*Opa1*) [41], microtubule cytoskeleton organization (*Wdpcp*) [42], and ion transport (*Letm1*) [43], for further investigation. We first conducted gene-specific m^6^A qPCR assays to validate the increased m^6^A levels of these four mRNAs in the cerebellum of KO mice (Fig. 6e). Subsequently, subcellular fractionation analysis of cerebellar cells was performed (Additional file 1: Figure S8f, g). Compared to WT mice cerebellum, all the four RNAs invariably exhibited increased abundance in the cytoplasm in the *Alkbh5* deficient cerebellum (Fig. 6f). Together, these data indicate that *Alkbh5* deficiency led to dysregulated RNA nuclear export in the cerebellum.

Given the role of m^6^A in regulating RNA stability, we also analyzed the gene expression changes resulting from *Alkbh5* deficiency. Among the 497 differentially expressed RNAs**,** 81 exhibited changed methylation and were enriched in functional pathways, including transport, cell division, and cell cycle control (Additional file 6: Table S8). For instance, the genes involved in cell cycle control, such as *Ddx11*, *Ccnb1*, and *Cbx1*, displayed a reduction in their RNA expression together with their reduced methylation. For genes associated with transport or metabolism, *Alkbh5* deficiency-induced RNA hypermethylation led to either a decrease (such as *Cacna2d3*, *Notch3*, and *Jam3*) or increase (such as *Slc6a20a*, *Dlk1*, *Slc2a4*, and *Ascl2*) in their RNA expression in the cerebellum (Additional file 1: Figure S8h).

Taken together, our data confirm that, by altering the original m^6^A level, *Alkbh5* deficiency disturbed the RNA metabolism of a subset of cell fate determination genes and thus led to defective cerebellar development in the mice exposed to hypobaric hypoxia.

## Discussion

Brain development is known to be regulated in a precise spatiotemporal manner. We have recently identified region-specific m^6^A with distinct methylation patterns and biological functions for the mouse cerebellum and cerebral cortex [36]. In the present study, we further demonstrate that developmentally regulated m^6^A methylation is one of the important regulatory factors for postnatal mouse cerebellar development. Temporal-specific methylation differs from continuous methylation in its methylation level, biological functions, and dynamic distribution of m^6^A marks. Furthermore, we uncovered that abnormal expression of m^6^A methyltransferases (METTL3) and demethylases (ALKBH5) caused imbalanced RNA methylation, which further led to defective cerebellar development. We also discovered that *Alkbh5* deficiency led to facilitated nuclear export of those hypermethylated RNAs.

Previous identified changes in genome-wide gene expression and epigenetic modifications throughout the whole cerebellar developmental processes have greatly improved our understanding of the mechanism related to mouse cerebellar development [34, 35, 44]. Given the role of m^6^A in fate determination of embryonic stem cells and mammalian cortical neurogenesis [16, 17, 31, 45], we hypothesized that m^6^A also participates in neuronal cell proliferation and differentiation in the mouse cerebellum. In this study, we show that a considerable number of RNAs in the mouse cerebellum exhibit developmentally regulated methylation. GO analysis implied that numerous genes regulating cell proliferation, differentiation, or metabolism acquire m^6^A marks in a temporal-specific manner, among which the expression of 839 RNAs was under tight regulation by their methylation status. Notably, we found that the number of differentially methylated RNAs is much higher than that of differentially expressed RNAs as observed during cerebellar development and in *Alkbh5* KO mice. We speculate that this is probably due to the additional functions of RNA methylation in RNA splicing, nuclear export, and protein translation [5]. For example, although the hypermethylated RNAs presented in Fig. 6e did not change in their expression levels, their nuclear export was enhanced by *Alkbh5* deficiency. Since it is the transcripts in the cytoplasmic pool that are utilized for RNA translation to protein and RNA degradation, appropriate m^6^A levels are necessary for maintaining RNA nuclear export in a balanced state. These results imply that the selective m^6^A marks, through their various modulatory effects on RNA processing, function as epitranscriptomic switches that either activate or suppress a series of physiological events during mouse cerebellar development. In line with our findings in the mouse cerebellum, dynamic changes in m^6^A RNA methylation were also observed during postnatal liver development in pig [46] and mouse spermatogenesis [22].

The dynamic RNA methylation levels in the developing cerebellum reveal how important it is to maintain the balance of RNA methylation in the developing mouse cerebellum, which is accomplished by both the m^6^A methyltransferases and demethylases. The morphological changes observed upon transient knockdown or overexpression confirm that METTL3 is involved in regulating cerebellar development. In addition to methyltransferase, RNA m^6^A demethylase is another important factor to maintain RNA methylation at appropriate levels. The important role of FTO in the mouse cerebellum was shown in a previous report using a *Fto*-knockout mouse model, which is characterized by a cerebellum that is significantly smaller than that in WT litter mates [28]. ALKBH5, another demethylase identified earlier, regulates spermatogenesis [20] and maintains tumorigenicity of glioblastoma stem-like cells [47]. However, its function in the brain remains unclear. Here we only observed defects in cerebellar development in *Alkbh5*-deficienct mice upon exposure to hypobaric hypoxia. It is likely that ALKBH5, FTO, and probably other undefined demethylases overlap in their demethylation activity and can partially substitute for each other’s functions under normal conditions [37]. On the other side, our results also reveal the distinct functions between ALKBH5 and FTO. In line with a previous report [9], we provide in vivo evidence that ALKBH5 regulates the nuclear export of the hypermethylated RNAs, while FTO regulates RNA splicing in a methylation-dependent manner [7]. In this study, we observed increased cell proliferation in the *Alkbh5*-deficienct mouse cerebellum exposed to hypobaric hypoxia. In contrast, *Fto* deficiency in adult mouse hippocampus led to reduced cell proliferation in adult neural stem cells [28]. The discrepancy in these results confirmed that RNA methylation constitutes a complex process and suggests that the regulation and function of m^6^A methylation in the mouse brain should be investigated in a more precise spatiotemporal-dependent manner.

In the WT mouse cerebellum at P7, the m^6^A modification selectively marked a group of RNAs responsible for cell cycle control and cell division. In *Alkbh5* KO mice exposed to hypobaric hypoxia, we detected many of these types of RNAs with aberrant methylation, which may further disturb RNA metabolism, such as RNA splicing, nuclear export, RNA decay, or translation. In agreement with these findings, we observed an increase in the numbers of mitotic cells in the cerebellum of *Alkbh5* KO mice upon exposure to hypobaric hypoxia. Mechanistically, we showed the hypermethylation and enhanced nuclear export of *Mphosph9*, which encodes a centrosomal protein related to cell division and cell cycle control [40]. Therefore, abnormal RNA methylation is likely one of the reasons resulting in dysregulated cell proliferation and cell cycle progression observed in the *Alkbh5*-deficient cerebellum. Consistent with our findings, deficiency in the m^6^A reader *Ythdf2* delayed DNA synthesis and mitotic processes in a zebrafish model [24]. In another study, deletion of *Mettl14* in embryonic mouse brain also led to prolonged cell cycle of cortical neural progenitors [31]. Together, these phenotypes provide strong evidence that balanced m^6^A levels are essential for cell cycle control. Moreover, we found that a higher number of RNAs were hypermethylated in *Alkbh5-*deficient cerebella of P7, which were originally methylated only in fully differentiated, mature neural cells in WT cerebellum. Such untimely and aberrant methylation finally appears to result in premature differentiation and aberrant neurogenesis in the cerebellum. This may explain the phenotypes of lower numbers of mature granule neurons in IGLs, together with disorganized Purkinje cells, and astrocytes. Generally, cell fate determination requires a burst of expression of a distinct group of genes at each specified stage, which is spatially and temporally regulated throughout the whole developmental process. Our results show that the positive or negative regulation of m^6^A marks on these cell fate-determining genes is important to drive the proper progression of postnatal cerebellar development.

Different from previous in vitro analysis showing that m^6^A marks are predominantly located near stop codons and 3′ UTRs, previous in vivo methylation analysis of mouse cerebellum and cerebral cortex identified the m^6^A marks in start codon regions as well as stop codons and 3′ UTRs [36]. Here, we identified similar patterns of m^6^A distribution throughout the process of postnatal cerebellar development. Importantly, we observed a significant change in the distribution of temporal-specific m^6^A marks from P7 to P60. We deduce that the distribution patterns of temporal-specific m^6^A peaks possibly reflect the difference in methylation sites between proliferating, immature and fully differentiated, mature neural cells. In the mouse cerebellum of P7, during which the majority of neural cells are actively proliferating, the temporal-specific m^6^A peaks were enriched in CDS, stop codons, and 3′ UTRs, which was similar to the results obtained from the m^6^A-seq analysis performed using cell lines [2, 3, 48]. In contrast, distribution of temporal-specific m^6^A peaks in start codon regions was most significant in mouse cerebellum at P60, which mainly consists of well-differentiated and mature neural cells. In support of our findings, m^6^A marks in start codon regions were also observed in the fully differentiated tissues from mouse cerebral cortex [36] (unpublished data), *A. thaliana* [49], rice [50], liver [46], and muscles [51]. Collectively, these data imply that the methylation in start codon regions occurs not only in mature neural cells of the mouse cerebellum, but also in many other types of fully differentiated cells in diverse organisms. We thus propose that the position of m^6^A marks included in the transcript may be an important factor that determines the functions mediated by m^6^A. Further investigation will be necessary to clarify the precise roles of m^6^A in different physiological processes. However, the m^6^A-IP-seq used in this study was unable to identify the exact position of the m^6^A marks. We cannot rule out the possibility that the m^6^A marks located in 5′ UTR may be misclassified into start codon regions for those poly(A) RNAs with a very short 5′ UTR. Thereby, single nucleotide-resolution m^6^A analysis [48, 52] should be utilized next to identify the exact positions of m^6^A marks, which will further enable exploration of the functions and regulatory mechanism of m^6^A deposited in different regions of the transcripts. In addition, the different patterns of m^6^A observed between cell lines and tissues indicates the complexity of in vivo temporal regulation of RNA methylation, suggesting the necessity to characterize RNA methylation in a context-dependent manner.

Gene regulation in the brain is characterized by its considerable cellular heterogeneity, including gene expression [53], DNA hydroxymethylation [34], and m^6^A RNA methylation [36]. Accordingly, we found that during cerebellar development, the expression of m^6^A writer and eraser genes in granule neurons and Purkinje neurons changed in opposite directions. Although the poly(A) RNAs analyzed in this study were isolated from a mixture of all neural cell types in the mouse cerebellum, the methylation profiles depicted here mainly reflect the nature of m^6^A modification in granule neurons as they constitute about 90% of the cerebellar neural cells [35]. For further insights, it will be important to elucidate the methylation profiles and related biological functions in Purkinje cells and glial cells, respectively. A more detailed, cell type-specific methylation analysis will be necessary to gain insights into the biological functions and developmental regulation of m^6^A modification in individual types of neural cells.

## Conclusions

Here, we describe the first m^6^A RNA methylation landscape during postnatal development of the mouse cerebellum and reveal the unique features of continuously and temporally specific methylation in the mouse cerebellum from P7 to P60. Our results indicate that temporal regulation of RNA m^6^A methylation, orchestrated by spatiotemporal expression of m^6^A writers and erasers, is essential in regulation of postnatal cerebellar development. We also provide the first in vivo evidence that ALKBH5-induced abnormal RNA methylation affects RNA nuclear export. Further studies will be necessary to uncover the detailed molecular mechanism of the regulation and biological functions of m^6^A during mouse cerebellar development. In addition, characterization of the role of m^6^A in the cerebellum will offer a new viewpoint to elucidate the mechanism of related neurological diseases.

## Methods

### Animal care

Wild-type C57BL/6 mice were purchased from Vital River company (Beijing, China). The *Alkbh5*-deficient mice were used and genotyped as described previously [9]. For all experiments performed here, equal numbers of male and female mice were included for analysis. All animal experiments and euthanasia were approved and performed in accordance with the guidelines of Animal Care and Use Committee of IBMS/PUMC. The IRB (Institutional Review Board) approval number is ACUC-A02–2014-001.

### Cell lines

Neuro2a cells and HEK293T cells were purchased from National Infrastructure of Cell Line Resource (Beijing, China). The cell lines have been authenticated and tested for mycoplasma contamination by the provider.

### Antibodies

Detailed information on the primary and secondary antibodies used in this study is listed in Additional file 1: Table S9.

### RNA isolation, poly(A) RNA purification, and m^6^A-IP

Mice were sacrificed by cervical dislocation and cerebellum was removed and immediately snap-frozen in liquid nitrogen. Total RNA was extracted using TriReagent (Sigma, T9424) according to the manufacturer’s instructions. In general, four mice of P7, four mice of P14, and three mice of P21 or P60 were used for one m^6^A-IP reaction according to the established protocol [54]. Briefly, poly(A) RNA was purified from total RNA using Poly(A)Purist™ MAG Kit (Thermo Fisher, AM1922). Next, poly(A) RNA was fragmented to 100-150 nucleotides (for m^6^A-seq) using RNA Fragmentation Reagents (Thermo Fisher, AM8740) as instructed by the manufacture. Fragmented poly(A) RNA (1 μg) was incubated with 2.5 μg of anti-N^6^-methyl-adenosine antibody at 4 °C for 4 h, followed by addition of Dynabeads™ Protein A for Immunoprecipitation (Thermo Fisher, 10002D). After incubation at 4 °C for 2 h, beads were extensively washed five times with IPP buffer (150 mM NaCl, 0.1% NP-40, 10 mM Tris-HCl, pH 7.4); immunoprecipitated RNA was recovered through elution with m^6^A nucleotide followed by ethanol precipitation.

For m^6^A-seq, a cDNA library was constructed using a KAPA Stranded mRNA-seq kit (Kapa Biosystems, KR0960) and subjected to next-generation sequencing on an Illumina Hiseq X Ten system. Input RNA before immunoprecipitation was applied to RNA-seq in parallel.

For gene-specific m^6^A-IP-qPCR, m^6^A-IP was performed using 3 μg of fragmented poly(A) RNA (300–500 nucleotides). Reverse transcription was carried out with an equal ratio of RNA from input and IP product by using ReverTra Ace® qPCR RT Master Mix (TOYOBO, FSQ-301). Quantitative real-time PCR was performed by using THUNDERBIRD™ SYBR® qPCR Mix (TOYOBO, QPS-201). *Gapdh* was used as a negative control. The primers used in this study are listed in Additional file 1: Table S10.

### Protein extraction and western blot analysis

Cerebella were dissected from mice of different ages and triturated in RIPA lysis buffer with protease inhibitor cocktail (Roche, 04693159001) and phosphatase inhibitors. Tissue lysate (60–80 μg) was subjected to 10% SDS-PAGE. After electrotransfer, the blots were blocked in 5% milk in Tris-buffered saline with Tween 20 (TBST) for 1 h at room temperature, then incubated at 4 °C overnight with the primary antibodies. The transferred membranes were incubated with appropriate HRP-conjugated secondary antibodies, exposed to enhanced chemiluminescence (ECL) solution, and visualized by gel image analysis system (Tanon, 5800).

### Histology and immunostaining analysis

After anesthesia with saturated tribromoethanol solution, mice were perfused with 4% paraformaldehyde; the whole brains were dissected and post-fixed in 4% paraformaldehyde for 24–48 h at 4 °C. For paraffin sections, the cerebellum was dehydrated with ethanol, clarified with xylene, and then embedded in paraffin. We used 4-μm-thick sections for hematoxylin and eosin (H&E) staining or immunohistochemistry analysis. For cryosections, the cerebella were dehydrated in 30% sucrose for more than 24 h, embedded in OCT compound and frozen at − 40 °C. We used 12-μm-thick sections for immunofluorescence analysis. Immunohistochemistry and immunofluorescence staining were performed according to standard protocols [55].

For BrdU incorporation analysis, the mice were intraperitoneally injected with BrdU (Sigma, B5002; final concentration 50 μg/g) and analyzed 2 hours after BrdU injection. Immunofluorescence analysis was performed as described above by using anti-BrdU antibody.

Images of H&E and immunohistochemical staining were recorded using a Panoramic MIDI II digital slide scanner (3D Histech). Results of immunofluorescence analysis were imaged with a confocal microscope (Zeiss, LSM780).

### Quantitative m^6^A level measurement using UHPLC-MS/MS

The absolute amount of m^6^A in the poly(A) RNA was measured as described before [36]. Briefly, 100 ng of poly(A) RNA was digested with nuclease P1 (Sigma) at 37 °C for 2 h, followed by treatment with calf intestine alkaline phosphatase (CIAP, Promega) at 37 °C for 2 h. The solution was filtered and analyzed using ultra-performance liquid chromatography and a triple-4 quadrupole mass spectrometer (AB SCIEX QTRAP 5500). m^6^A level in each sample was calculated by comparing with the standard curve obtained from pure nucleoside standards loaded simultaneously. The ratio of m^6^A to A was calculated to obtain the global methylation level.

### Plasmid construction

The lentiviral overexpression vectors used in this study include pLV-EF1a-EGFP(2A) Puro, pH 1, and pH 2 vectors (kindly provided by Prof. Kaili Ma). cDNA of mouse *Mettl3* was cloned into pLV-EF1a-EGFP(2A) Puro vector digested with EcoR I and BamH I restriction enzymes. The overexpression efficiency of lentivirus was verified in Neuro2a cells by western blotting. The primers used were: forward, 5′-**cg**gaattc ATGTCGGACACGTGGAG-3′; reverse, 5′- **cg**ggatccCGCTATAAATTCTTAGG-3′.

The lentiviral knockdown system used in this study includes pLL3.7, pRSV-rev, pMDLg/pRRE, and pCMV-VSV-G (kindly provided by Prof. Qi Xu). shRNA of mouse *Mettl3* was cloned into pLL3.7 vector between HpaI and XhoI restriction enzyme sites. Two sets of shRNAs were used for each gene and were evaluated for their knockdown efficiency in Neuro2a cells via western blot analysis. The shRNA with higher efficiency was chosen for subsequent in vivo virus infection of mouse cerebellum. The oligos of *Mettl3* shRNA were: 1, 5′-aac**c**GCACACTGATGAATCTTTAGGTtcaagag ACCTAAAGATTCATCAGTGTGC**ttttt**c-3′; 2, 5′-aac**c**CTGCAAATATGTTCACTATGAtcaagag TCATAGTGAACATATTTGCAG**ttttt**c-3′. The negative control containing scrambled shRNA was 5′-aac**c**TTCTCCGAACGTGTCACGTtcaagagACGTGACACGTTCGGAGAA**ttttt**c-3′.

The plasmids generated here were written in short as OE, sh-1, sh-2.

### Lentivirus infection of mouse cerebellum

All lentivirus vectors were purified and amplified using QIAGEN Plasmid maxi kit (Qiagen, number 12163). 293 T cells were co-transfected with a mixture of lentiviral plasmids (including 17.5 μg of pLV-derived plasmid, 13.125 μg of pH 1, 4.375 μg of pH 2 for overexpression system; 14 μg of pLL3.7-derived plasmid, 7 μg of pMDLg/pRRE, 7 μg of pCMV-VSV-G, 7 μg of pRSV-Rev for knockdown system) using DNA transfection reagent (Neofect, TF20121201). Ssupernatants were collected 48 h after transfection and concentrated by ultracentrifugation. Titration of lentivirus was carried out by transducing 293 T cells in tenfold serial dilution. Generally the lentivirus used in this study had a titer of 1 × 10^8^ to 1 × 10^10^ TU/ml.

In vivo lentivirus infection was performed as previously described with minor modification [56]. Briefly, wild-type C57BL/6 mice (P7) were anesthetized on ice and then injected with a mixture of lentivirus (2 μl), polybrene (800 ng/μl), and fastgreen (0.1%) into the interlobular space between lobules V and VI. Pups were revived at 37 °C and then returned to original cages. After 7 (for overexpression) or 10 (for knockdown) days post-lentivirus infection, immunofluorescence analysis was performed as described above to detect the phenotypes of cerebellum upon lentivirus infection.

### Hypobaric hypoxia treatment of mice

WT or *Alkbh5* KO mice of P5, together with their mothers, were placed into a hypobaric hypoxic chamber and exposed to a simulated atmospheric pressure of 10.6 kPa (377 mmHg), which was equivalent to an altitude of 5500 m. All animals were kept at constant temperature (25–30 °C) on a daily light schedule of 12 h of light vs. dark with normal activity. The hypobaric hypoxic condition was maintained and monitored continuously with the sensor inside the chamber. Wild-type and *Alkbh5*^−/−^ pups were dissected for phenotype analysis after incubation in the hypobaric chamber for 48 h. To examine the phenotypes of hypoxia-treated mice at later developmental stages, the WT and KO mice were returned to normoxic conditions together with their mothers and raised for another 7 days before dissection for phenotype analysis.

### Magnetic resonance imaging analysis

Mice were anaesthetized with 0.75% amobarbital before analysis. Mouse brain MRI was performed on a 7.0 T BioClinscan Animal MRI System (Bruker, Germany) with Siemens manipulating software. T2-weighted images of the whole brain (including the sagittal, coronal, and transverse views) were acquired with a 2D-TSE (2D-turbo spin-echo) sequence. The scanning time for each mouse was about 10 min. The scanning parameters were as follows: sagittal view, TR = 2360 ms, TE = 41 ms, number of averages = 1, 15 axial slices with a slice thickness (ST) of 0.7 mm, a field of view (FOV) of 3.52 cm × 3.52 cm, and a matrix of 320 × 320 were positioned over the whole brain with a pixel spacing of 0.11 mm; coronal view, TR = 3510 ms, TE = 53 ms, number of averages = 1, axial slices = 19, ST = 0.5 mm, image matrix size = 320 × 384, pixel spacing = 0.078 mm, FOV 2.50 cm × 3.00 cm; transverse view, TR = 3380 ms, TE = 41 ms, number of averages = 1, axial slices = 21, ST = 0.7 mm, image matrix size = 320 × 320, pixel spacing = 0.094 mm, FOV 2.82 cm × 2.82 cm. Then the signals of different brain regions were measured and compared using a RadiAnt DICOM Viewer.

### Subcellular fractionation analysis of RNA

Fresh cerebella of WT and KO mice at P7 were minced and incubated in Digestion Solution (30 U/mL papain, 240 μg/mL cystein, 400 μg/mL DNAse I type IV) at 37 °C for 1 h. The reaction was stopped by adding Ovomuccoid inhibitor solution (1125 μg/mL Ovomuccoid trypsine inhibitor, 525 μg/mL BSA, 400 μg/mL DNase I type IV) and incubation at 37 °C for 4 min. After purification, cerebellar cells were subjected to cellular fractionation using a PARIS™ Kit (Thermo Fisher, AM1921) according to the manufacturer’s protocol. Nuclear and cytoplasmic RNAs (120 ng) were reverse transcribed using ReverTra Ace® qPCR RT Master Mix (TOYOBO, FSQ-301). Quantitative real-time PCR was performed using THUNDERBIRD™ SYBR® qPCR Mix (TOYOBO, QPS-201). *Nd1* was used as a cytoplasmic marker while *U1* was used as a nuclear marker. The primers used in this study are listed in Additional file 1: Table S10.

### RNA-seq data processing and reads mapped

For each sample, single-end reads were used for bioinformatics analysis. Quality control of raw data was done using FastQC software (version 0.10.1). Sequencing data were preprocessed with in-house Perl scripts using the following criteria: 1) adaptor sequence was removed by finding the sequence AGATCGGAAG with at most two mismatched bases; 2) bases with low quality score (< 20) were trimmed off from the 3′ end; 3) reads with length longer than 70 nucleotides and more than 70% bases with quality score > 25 were retained. These high-quality reads were mapped against the mouse genome (mm10) allowing up to two mismatches using TopHat software (version 2.0.13) [57, 58]. Only uniquely mapped reads were kept for the subsequent analysis.

### RNA expression analysis

The FPKM (fragments per kilobase of transcript per million mapped reads) values of RNAs in each sample were calculated using Cufflinks toolkit (version 2.0.2) [59]. Only the transcripts with FPKM value > 0.2 were considered as expressed transcripts [60]. The differentially expressed RNAs (DERs, *P* < 0.05) between any two samples were identified using Cuffdiff. The normalized FPKM value for each sample was used for clustering using the KMC method of MeV software with the following parameter settings: 1) Pearson correlation clustering; 2) K-means clustering for six clusters; 3) other parameters set as defaults [61].

To verify the reliability of the DERs, we performed a parallel differential expression analysis by STAR/edgeR packages. High quality reads in each sample were mapped to the mouse genome (mm10) using STAR software and the uniquely mapped reads were kept for the subsequent analysis [62, 63]. HTSeq software [64] was utilized to calculate the read count of RNAs and edgeR [65] was applied to identify the DERs (*P* < 0.05). All software applications were run with default parameters.

### m^6^A peak calling and motif analysis

RNA m^6^A-modified regions, also called m^6^A peaks, were identified using exomePeak software (version 2.7.0) with FDR (false discovery rate) < 0.05 [66, 67]. The consensus sequence motifs enriched in m^6^A peaks were identified by HOMER [68]. For further comparison of m^6^A modification between samples, we used coverageBed of BEDToods (version 2.26.0) [69] with the “-s”, “-splited”, “-counts”, and “-F 0.50” parameters to calculate the read count of each peak. The “IP FPKM”, “input FPKM”, and “Enrichment score” of peaks were calculated as following methods:

1 $$ {\mathrm{a}}_{\mathrm{i},\mathrm{j}}=\left({\mathrm{A}}_{\mathrm{i},\mathrm{j}}\ \mathrm{x}\ {10}^9\right)/\left({\mathrm{B}}_{\mathrm{j}}\ \mathrm{x}\ {\mathrm{C}}_{\mathrm{i},\mathrm{j}}\right) $$

2 $$ {\mathrm{b}}_{\mathrm{i},\mathrm{j}}=\left({\mathrm{D}}_{\mathrm{i},\mathrm{j}}\ \mathrm{x}\ {10}^9\right)/\left({\mathrm{E}}_{\mathrm{j}}\ \mathrm{x}\ {\mathrm{C}}_{\mathrm{i},\mathrm{j}}\right) $$

3 $$ {\mathrm{c}}_{\mathrm{i},\mathrm{j}}={\mathrm{a}}_{\mathrm{i},\mathrm{j}}/{\mathrm{b}}_{\mathrm{i},\mathrm{j}} $$

where a_i,j_ denotes the peak “IP FPKM” value of the *i*th methylation peak in the IP sample from the *j*th biological sample; A_i,j_ denotes the total reads mapped to the *i*th methylation peak in the IP sample from the *j*th biological sample; B_j_ denotes the total unique reads mapped to the mouse reference (mm10) in the IP sample from the *j*th biological sample; C_i,j_ denotes the length (base) of the *i*th methylation peak in the IP sample from the *j*th biological sample; b_i,j_ denotes the peak “input FPKM” value of the *i*th methylation peak in the input sample from the *j*th biological sample; D_i,j_ denotes the total reads mapped to the *i*th methylation peak in the input sample from the *j*th biological sample; E_j_ denotes the total unique reads mapped to the mouse reference (mm10) in the input sample from the *j*th biological sample; c_i,j_ denotes the peak “enrichment score” value of the *i*th methylation peak in the IP/input sample from the *j*th biological sample.

M^6^A peaks that satisfied 1) exomePeak FDR value < 0.05, 2) m^6^A peak “IP FPKM” value > 1, and 3) m^6^A peak “enrichment score” value > 1.5 were used for further comparative analysis. The continuously methylated RNAs (CMRs) were defined as RNAs containing at least one m^6^A peak in all the four samples, while the temporal-specific methylated RNAs (SMRs) were the RNAs with m^6^A modification only in one sample, and m^6^A peaks of SMRs were defined as specific m^6^A peaks.

By merging all the m^6^A peaks of four developmental stages using mergeBed of BEDTools with “-s”, “-c”, and “-o” parameters, we identified “ON” or “OFF” RNA m^6^A switches during cerebellar development. All m^6^A peaks absent in the former stage but present in the later stage were called “ON” m^6^A switches, while all m^6^A peaks displaying changes in the opposite direction were called “OFF” m^6^A switches. In addition, the differentially methylated RNAs (DMRs) between wild-type and *Alkbh5* knockout mice were further identified using exomePeak software with default parameters, and high-confidence DMR m^6^A peaks matching the criteria of FDR < 0.05 and log2 (fold change) > 1 or < − 1 were selected for further analysis.

For validation analysis of the distribution patterns of stage-specific m^6^A peaks which were obtained by using the exomePeak software, peak calling analysis was performed in parallel using the MACS2 software for the P7 and P60 samples with the default parameters [54, 70].

The integrative genomics viewer (IGV) tool was used for visualization of m^6^A peaks along the whole transcript [71, 72]. The heatmaps of the enrichment score for m^6^A peaks were plotted using the pheatmap package in R [73].

### Characterization of m^6^A peak distribution patterns

Distribution of m^6^A peaks along mRNAs were determined as previously described with minor revision [3, 18]. To characterize the distribution patterns of m^6^A peaks, a reference mouse transcriptome was built using the longest transcript of each gene. Next, each of the 5′ UTR, CDS, and 3′ UTR regions were split into 100 bins with equal length. The percentage of m^6^A peaks in each bin was calculated to represent the occupancy of m^6^A peaks along the whole transcripts.

We further counted the number of m^6^A peaks in the 5′ UTR, start codon region, CDS, stop codon region and 3′ UTR, respectively. Specifically, a 300-nucleotide region centered on start codons or stop codons was defined as start codon regions or stop codon regions [36].

### Correlation analysis between RNA m^6^A methylation level and RNA expression level

The global clustering analysis of RNA methylation levels (enrichment score) and expression levels (FPKM) among the four stages was performed using Cluster 3.0 software. The enrichment scores and FPKM of each RNA in each sample were adjusted with “mean” parameters in “Normalize genes” and “Center genes”. The hierarchical clustering method was employed using “Complete linkage”. The clustered heatmap figure was generated using TreeView-1.1.6r4-win software.

In order to obtain a dynamic view of the regulatory effect of RNA m^6^A methylation on RNA abundance, we calculated the correlation coefficients and the associated *P* value between the enrichment scores of m^6^A peaks and FPKM of methylated RNAs at the four stages using Pearson test in R package. High-confidence regulatory gene pairs were defined by the correlation coefficients (|r| > 0.95) with *P* value < 0.05. Cluster analysis was performed for those positively (*r* > 0.95) and negatively (*r* < − 0.95) correlated RNAs using MeV software, respectively. The MeV cluster results were integrated and plotted using R programming language.

### GO analysis

The DAVID tool [74] was used for GO analysis by applying default parameters, except that only those transcripts fulfilling the condition of FPKM > 0.2 in the input samples were set as background. Either bubble plots or column plots were generated based on the enriched GO terms using the ggplot2 in R package [75]. Color intensity indicates the value of −log_10_(*P* value), while the length of columns or the size of the circles indicate the gene counts. A full list of all selected terms of the biological process, cellular components, and molecular functions category are provided in Supplemental tables (Additional file 2-4: Table S4-S6; Additional file 6: Table S8).

### Kyoto Encyclopedia of Genes and Genomes analysis

All modified genes were annotated to Kyoto Encyclopedia of Genes and Genomes (KEGG) pathways using KAAS (KEGG Automatic Annotation Server) [76], and the enriched KEGG pathways for the genes encoded by P7 or P60 temporal-specific SMRs or CMRs were identified using Fisher’s exact test (Additional file 4: Table S6) [77].

### Quantification and statistical analysis

The differences in distribution for m^6^A enrichment score and log_2_ FPKM between samples were detected by Wilcoxon test. Pearson test was used to perform correlation analysis. All statistical analysis and graphs of results in Figs. 3d, 4d, 5a, 5c–5f and 6f, and Additional file 1: Figures S5c, S6a, S7d, S7e, S8a and S8g were assessed using two-tailed unpaired Student’s *t*-test and performed using GraphPad Prism 6.0 software. Results are presented as mean ± s.e.m. Immunoreactive cells in 3–5 randomly selected lobules of one cerebellum were counted using StataQuest 5.0 software. For each subject, data were collected from five to seven mice. For each mouse, data were obtained from the immunostaining results of two to three near-midline slices.

## Additional files

Additional file 1: **Figure S1.** Dynamic RNA methylation in the developing mouse cerebellum. **Figure S2.** Comparison between continuous and temporal-specific methylation during mouse cerebellar development. **Figure S3.** Comparison of RNA methylation between P7 and P60 based on m^6^A peaks identified using MACS2. **Figure S4.** Correlation between RNA methylation and gene expression during cerebellar development. **Figure S5.** Morphology analysis of mouse cerebellum upon lentivirus infection for *Mettl3* overexpression. **Figure S6.** Phenotype analysis of the cerebellum in the WT and KO mice under normoxic condition. **Figure S7.** Morphology analysis of the cerebellum in WT and KO mice exposed to hypobaric hypoxia and normoxia successively. **Figure S8.** Dysregulated RNA methylation resulting from *Alkbh5* deficiency in mouse cerebellum exposed to hypobaric hypoxia. **Table S1.** Data quality and processing information of m^6^A-seq of poly(A) RNA from wild-type mouse cerebellum (P7, P14, P21, and P60), the cerebellum of wild-type (WT) and *Alkbh5* knockout (KO) mice exposed to hypobaric hypoxia (P7). **Table S2.** Statistics of m^6^A peaks and expressed RNAs in wild-type mouse cerebellum (P7, P14, P21, and P60), the cerebellum of wild-type (WT) and *Alkbh5* knockout (KO) mice exposed to hypobaric hypoxia (P7). **Table S3.** Numbers of m^6^A peaks located in different regions of mRNA transcripts in wild-type mouse cerebellum at P7, P14, P21, and P60. **Table S9.** List of antibodies and their applications used in this study. **Table S10.** List of primers for RT-qPCR used in this study. (PDF 14643 kb)

Additional file 2: **Table S4.** GO analysis of genes containing m^6^A ON and OFF switches during mouse cerebellar development. (XLSX 564 kb)

Additional file 3: **Table S5.** GO analysis of genes encoded by the CMRs at P7 and P60, and SMRs at the four developmental stages. (XLSX 128 kb)

Additional file 4: **Table S6.** GO and KEGG pathway enrichment analysis of the genes encoded by SMRs and CMRs compared between the m^6^A peaks at P7 and P60 that were identified by using MACS2 software. (XLSX 303 kb)

Additional file 5: **Table S7** List of the 839 RNAs with strong positive or negative correlation between their methylation levels and expression levels. (XLSX 82 kb)

Additional file 6: **Table S8** GO analysis of the genes with altered m^6^A levels between wild-type and *Alkbh5-*deficient mice exposed to hypobaric hypoxia. (XLSX 83 kb)

## Abbreviations

BP: Biological processes
CC: Cellular components
CDS: Coding sequence
CMRs: Continuously methylated RNAs
DMRs: Differentially methylated RNAs
ECL: Enhanced Chemiluminescence
EGL: External granule cell layer
FDR: False discovery rate
GO: Gene Ontology
HE: Hematoxylin-eosin staining
IGL: Inner granule cell layer
KEGG: Kyoto Encyclopedia of Genes and Genomes
KO: *Alkbh5*-knockout
m^6^A: N^6^-methyladenosine
P7: Postnatal day 7
PCL: Purkinje cell layer
PH3: Phospho-histone 3
SMRs: Specifically methylated RNAs
UHPLC-MS/MS: Ultra high performance liquid chromatography-mass spectrum/mass spectrum analysis
TBST: Tris Buffered Saline with Tween 20
WT: Wild-type
