# Supplementary material for: RNA m6A methylation participates in regulation of postnatal development of the mouse cerebellum
Source: Genome Biol. 2018 May 31;19:68. doi: 10.1186/s13059-018-1435-z (PMC5984455; doi:10.1186/s13059-018-1435-z)
Supplement: Supplementary file 1 — Figure S1. Dynamic RNA methylation in the developing mouse cerebellum. Figure S2. Comparison between continuous and temporal-specific methylation during mouse cerebellar development. Figure S3. Comparison of RNA methylation between P7 and P60 based on m6A peaks identified using MACS2. Figure S4. Correlation between RNA methylation and gene expression during cerebellar development. Figure S5. Morphology analysis of mouse cerebellum upon lentivirus infection for Mettl3 overexpression. Figure S6. Phenotype analysis of the cerebellum in the WT and KO mice under normoxic condition. Figure S7. Morphology analysis of the cerebellum in WT and KO mice exposed to hypobaric hypoxia and normoxia successively. Figure S8. Dysregulated RNA methylation resulting from Alkbh5 deficiency in mouse cerebellum exposed to hypobaric hypoxia. Table S1. Data quality and processing information of m6A-seq of poly(A) RNA from wild-type mouse cerebellum (P7, P14, P21, and P60), the cerebellum of wild-type (WT) and Alkbh5 knockout (KO) mice exposed to hypobaric hypoxia (P7). Table S2. Statistics of m6A peaks and expressed RNAs in wild-type mouse cerebellum (P7, P14, P21, and P60), the cerebellum of wild-type (WT) and Alkbh5 knockout (KO) mice exposed to hypobaric hypoxia (P7). Table S3. Numbers of m6A peaks located in different regions of mRNA transcripts in wild-type mouse cerebellum at P7, P14, P21, and P60. Table S9. List of antibodies and their applications used in this study. Table S10. List of primers for RT-qPCR used in this study. (PDF 14643 kb) [file 13059_2018_1435_MOESM1_ESM.pdf]

Additional file 1

RNA m^{6}A Methylation Participates in Regulation of Postnatal Development of the Mouse Cerebellum

Chunhui Ma, Mengqi Chang, Hongyi Lv, Zhi-Wei Zhang, Weilong Zhang, Xue He, Gaolang Wu, Shunli Zhao, Yao Zhang, Di Wang, Xufei Teng, Chunying Liu, Qing Li, Arne Klungland, Yamei Niu, Shuhui Song, Wei-Min Tong

Figure S1. Dynamic RNA methylation in the developing mouse cerebellum.

Figure S2. Comparison between continuous and temporal-specific methylation during mouse cerebellar development.

Figure S3. Comparison of RNA methylation between P7 and P60 based on m^{6}A peaks identified using MACS2.

Figure S4. Correlation between RNA methylation and gene expression during cerebellar development.

Figure S5. Morphology analysis of mouse cerebellum upon lentivirus infection for *Mettl3* overexpression.

Figure S6. Phenotype analysis of the cerebellum in the WT and KO mice under normoxic condition.

Figure S7. Morphology analysis of the cerebellum in WT and KO mice exposed to hypobaric hypoxia and normoxia successively.

Figure S8. Dysregulated RNA methylation resulting from *Alkbh5*-deficiency in mouse cerebellum exposed to hypobaric hypoxia.

Table S1. Data quality and processing information of m^{6}A-seq of poly (A) RNA from wild-type mouse cerebellum (P7, P14, P21 and P60), the cerebellum of wild-type (WT) and *Alkbh5*-knockout (KO) mice exposed to hypobaric hypoxia (P7).

Table S2. Statistics of m^{6}A peaks and expressed RNAs in wild-type mouse cerebellum (P7, P14, P21 and P60), the cerebellum of wild-type (WT) and *Alkbh5*-knockout (KO) mice exposed to hypobaric hypoxia (P7).

Table S3. Numbers of m^{6}A peaks located in different regions of mRNA transcripts in wild-type mouse cerebellum of P7, P14, P21 and P60.

Table S9. List of antibodies and their applications used in this study.

Table S10. List of primers for RT-qPCR used in this study.

Ma *et al*., Figure S1, related to Figure 1

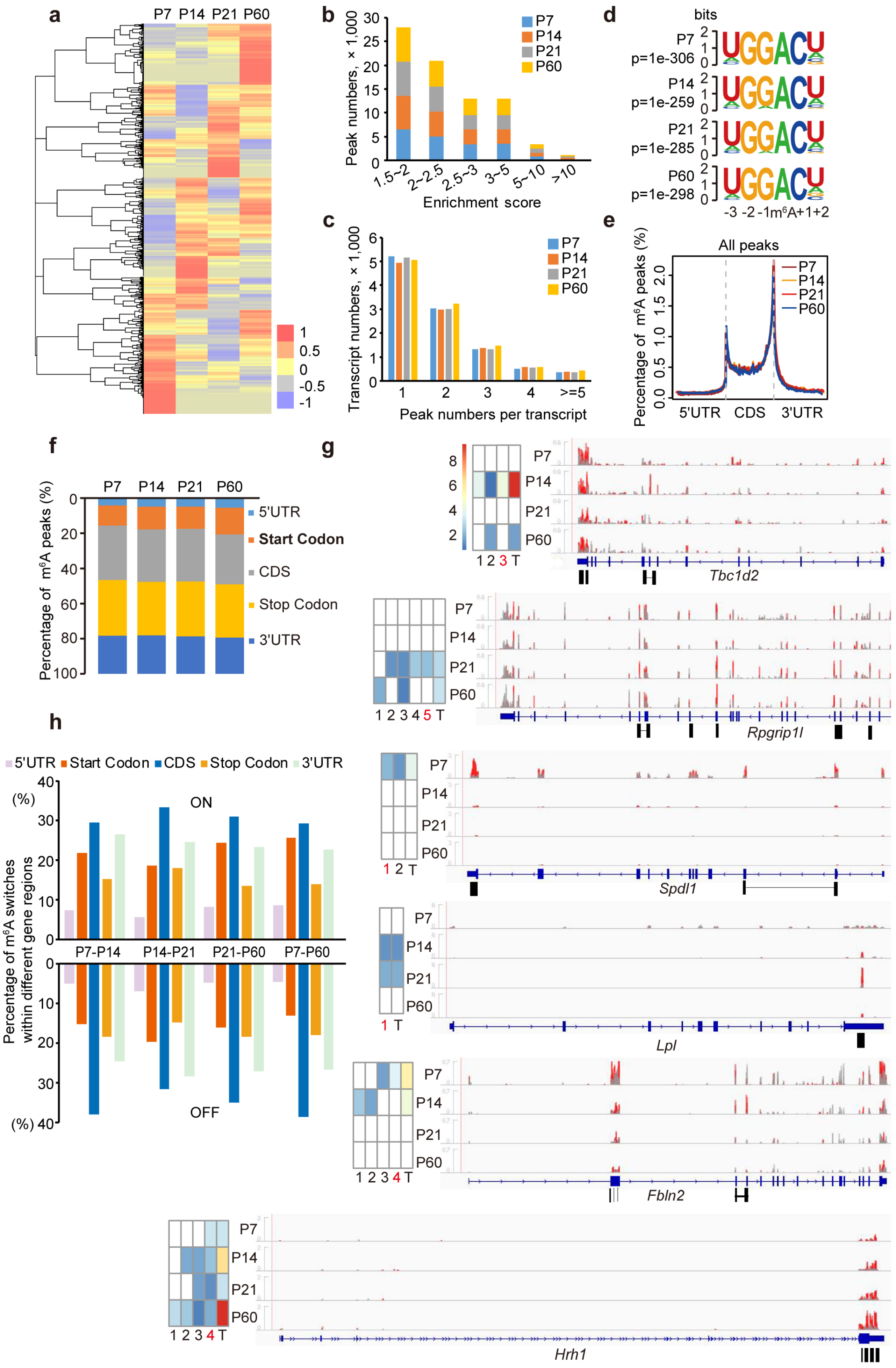

Additional file 1: Figure S1 (Related to Figure 1) Dynamic RNA methylation in the developing mouse cerebellum. **a** Heatmap representing enrichment scores for all methylated RNAs in mouse cerebellum from P7 to P60. **b** Numbers of m^6A peaks with different enrichment scores detected in the poly(A) RNAs of mouse cerebellum from P7 to P60. **c** Numbers of transcripts containing different numbers of m^6A peaks per transcript in the mouse cerebellum of the four stages. **d** Consensus sequence identified within m^6A peaks of the poly(A) RNAs of mouse cerebellum from P7 to P60. **e** Distribution of all m^6A peaks across the whole mRNA transcripts as visualized by the percentage of m^6A peaks falling in each transcript segment. **f** Column charts showing the percentage of all m^6A peaks falling in each transcript segment. **g** Visualization of the methylation profiles of RNAs containing ON or OFF switches from P7 to P60 by using heatmap (left panel) and IGV plot (right panel). Enrichment scores of each peak and the whole transcript (T) are shown in the heatmap. Peaks are numbered according to the transcription direction shown in IGV, and the peaks shown in Fig.1b are highlighted in red color. The tracks in IGV plots represent the reads density in IP (red) and INPUT (grey) samples for specific developmental stage. Positions of m^6A peaks are indicated by black lines under the transcript. **h** Column charts showing the percentage of m^6A ON/OFF switches falling in each transcript segment.

Ma *et al.*, Figure S2, related to Figure 2

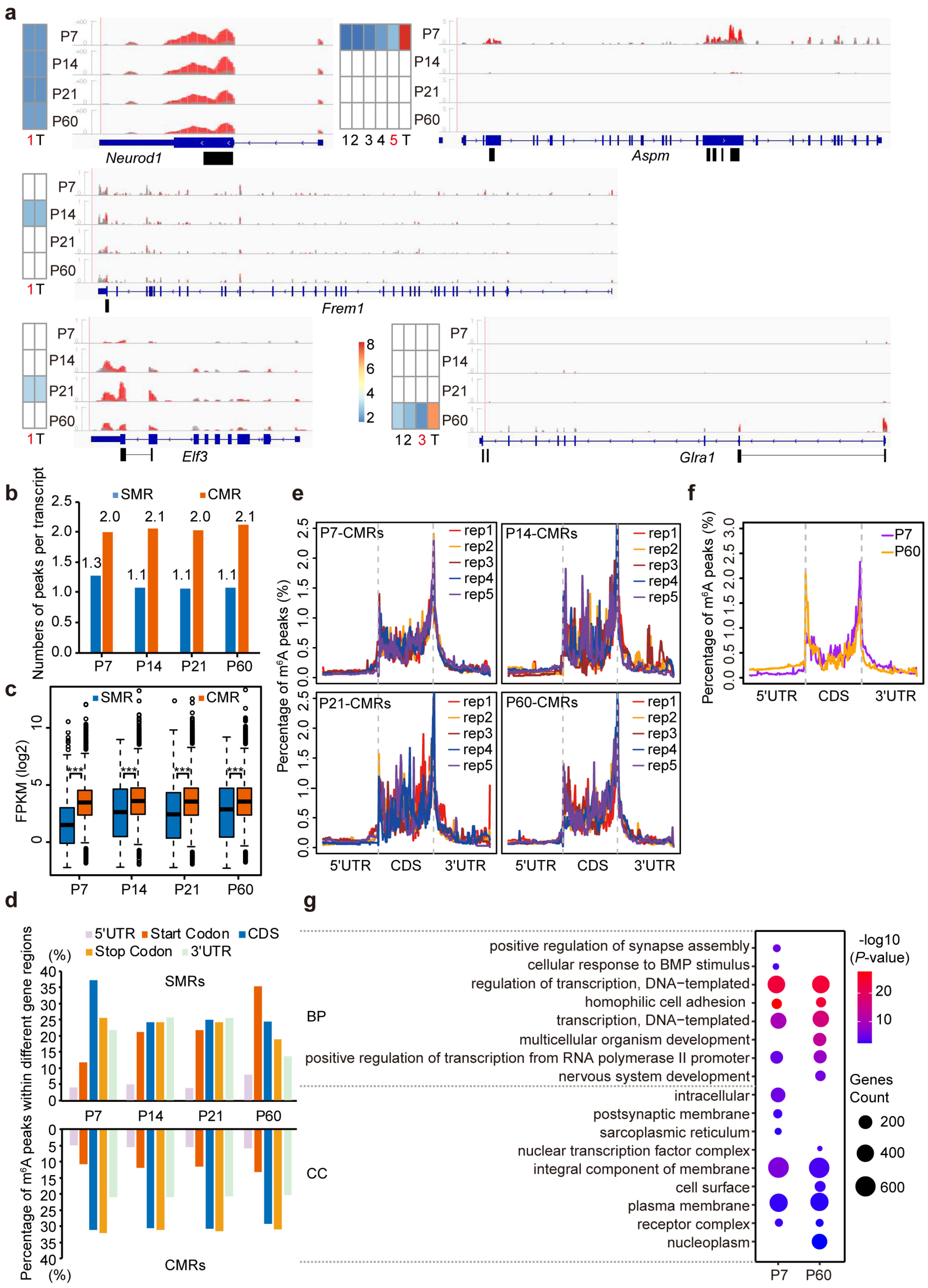

Additional file 1: Figure S2 (Related to Figure 2) Comparison between continuous and temporal-specific methylation during mouse cerebellar development. **a** Visualization of the methylation profiles of CMRs and SMRs as shown in **Fig. 2b** by heatmaps (left panel, with each peak per column numbered with Arabic numbers plus one column representing the whole transcript which short by “T”) and IGV plots (right panel, each track represents the reads density in IP and INPUT samples for specific developmental stage). **b** Column charts showing the average peak numbers per transcript of SMRs and CMRs. CMR, continuously methylated RNA; SMR, specifically methylated RNA. **c** Boxplots showing the relative expression levels of SMRs and CMRs. *** $P < 0.001$ by Wilcoxon test. **d** Percentage of m^6A peaks in SMRs and CMRs located in different regions across the whole mRNA transcripts. **e** Enrichment of m^6A peaks in CMRs across the whole mRNA transcripts in the four stages as analyzed by using the same numbers of peaks as those of SMRs peaks. The analysis was repeated five times by using randomly extracted peaks. **f** Distribution of P7- and P60-specific m^6A peaks along the whole mRNA transcripts. The temporal specific peaks shown here were identified by peaks comparison between P7 and P60 directly, which were called by using exomePeak software. **g** Most impacted GO biological processes and cellular components terms of CMRs at P7 and P60. The CMRs containing the top 3000 m^6A peaks in each sample were subjected to GO analysis.

Ma *et al.*, Figure S3, related to Figure 2

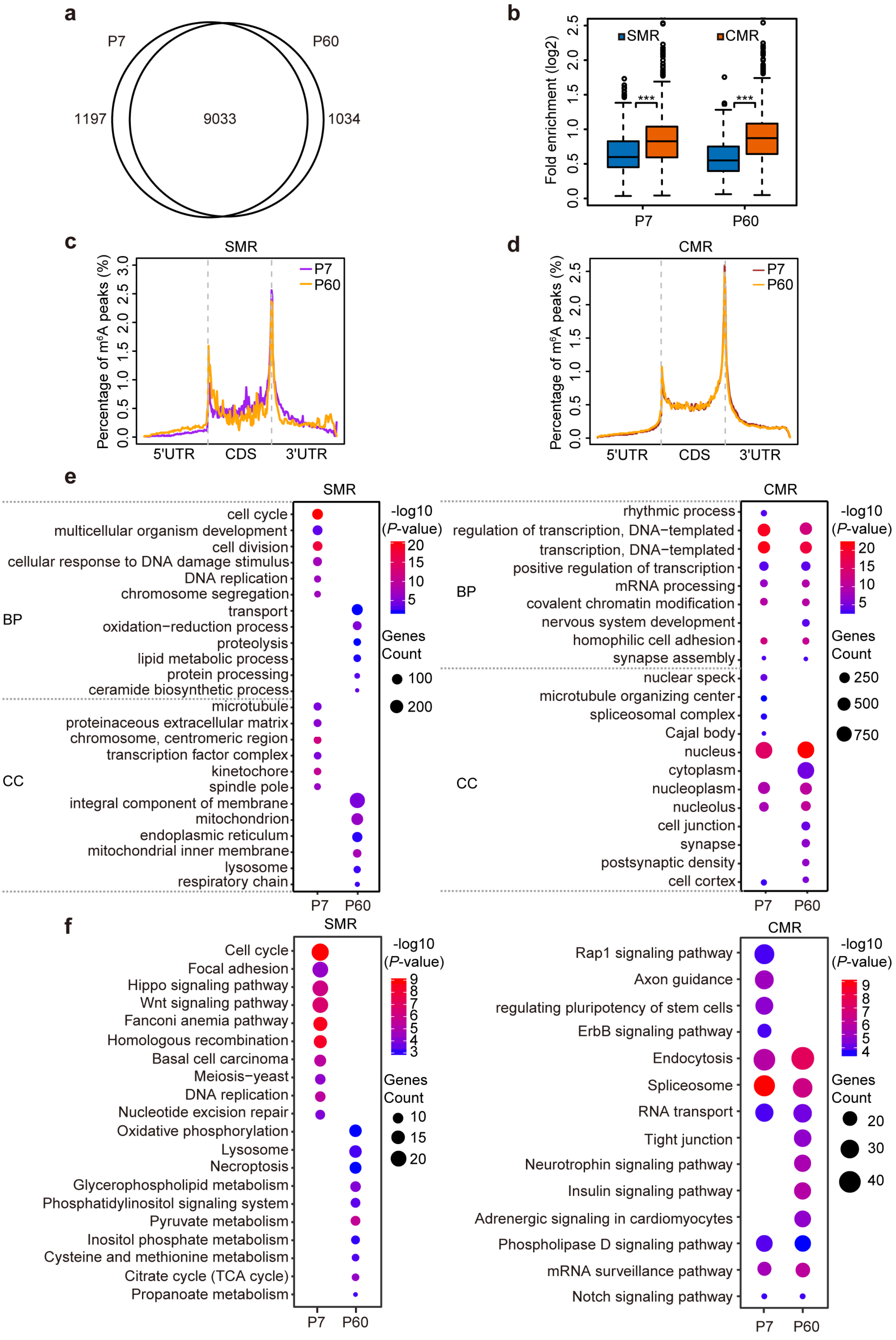

Additional file 1: Figure S3 (Related to Figure 2) Comparison of RNA methylation between P7 and P60 based on m^6A peaks identified using MACS2. **a** Venn diagram showing the numbers and relationship of methylated poly(A) RNAs identified by MACS2 in the mouse cerebellum of P7 and P60. **b** Box plots showing the relative methylation levels of SMRs and CMRs as evaluated by the enrichment scores of m^6A peaks. SMR, specifically methylated RNA; CMR, continuously methylated RNA. *** $P < 0.001$ by Wilcoxon test. **c, d** Distribution of m^6A peaks in the SMRs **(c)** and CMRs **(d)** along the whole mRNA transcripts. **e, f** Most impacted GO **(e)** and KEGG **(f)** pathways annotated to the SMRs and CMRs by comparing P7 and P60 directly.

Ma *et al*., Figure S4, related to Figure 2

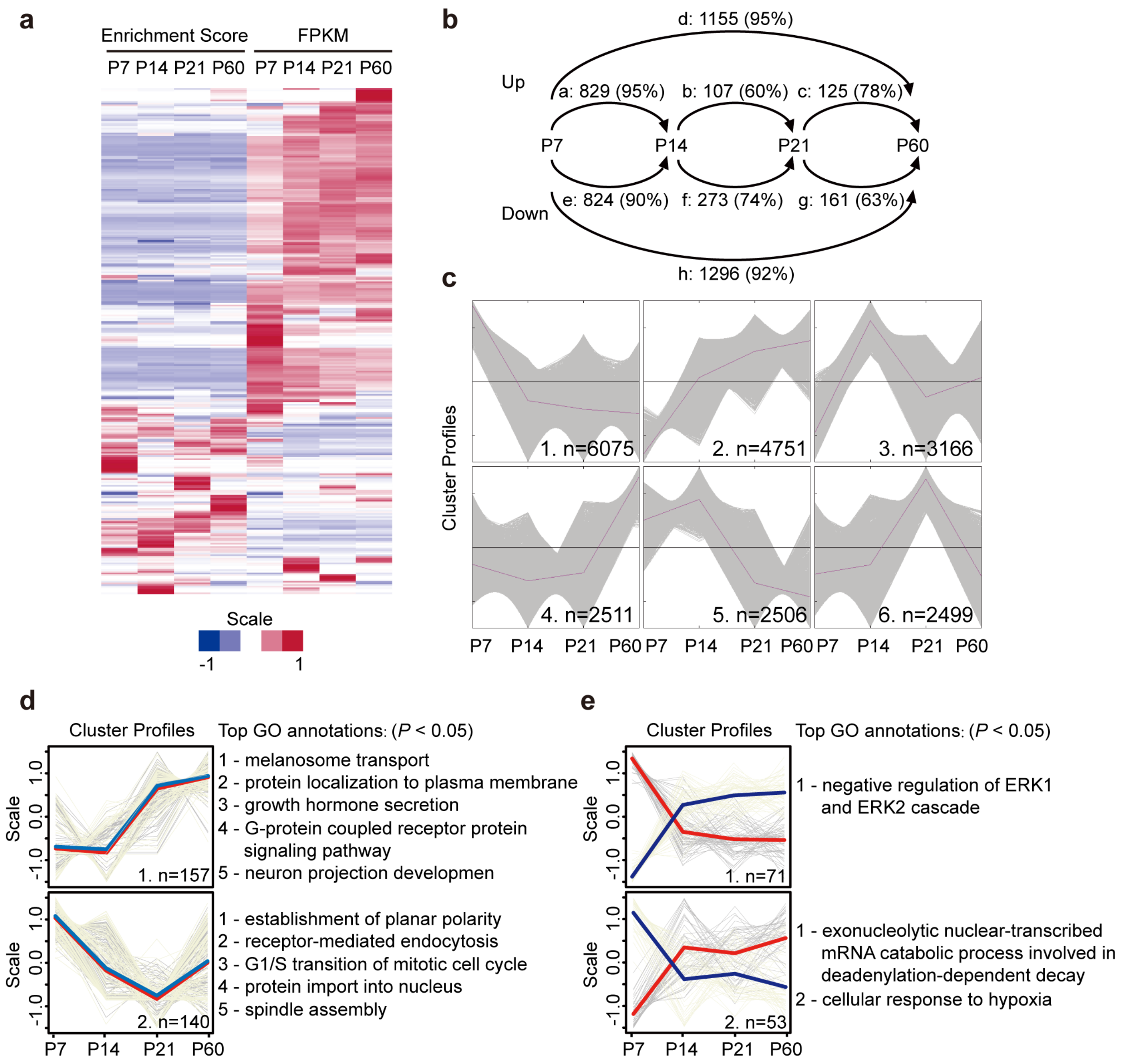

Additional file 1: Figure S4 (Related to Figure 2) Correlation between RNA methylation and gene expression during cerebellar development. **a** Heatmap showing RNA methylation levels (Enrichment Score) and gene expression levels (FPKM) of all expressed RNAs in mouse cerebellum from P7 to P60. **b** Numbers of differentially expressed genes (Up and Down) between P7, P14, P21 and P60 cerebella pairwise comparisons. The percentage of DEGs that could be validated by another parallel DEG analysis package (STAR/edgeR) is included in the parentheses. **c** Cluster analysis of all expressed RNAs (FPKM > 0.2) in the four developmental stages. Numbers of RNAs in each cluster are included. **d, e** Two clusters of RNAs possessing very high positive **(d)** (Pearson correlation coefficient > 0.95) or negative **(e)** (Pearson correlation coefficient < -0.95) correlation between methylation and expression levels from P7 to P60. The RNA expression profiles are displayed in beige, while the methylation profiles are displayed in grey. Numbers of RNAs in each cluster are included. Red line marks average methylation profiles, and blue line marks average expression profiles in each cluster. The most enriched GO pathways among these RNAs are listed. $P < 0.05$.

Ma *et al.*, Figure S5, related to Figure 4

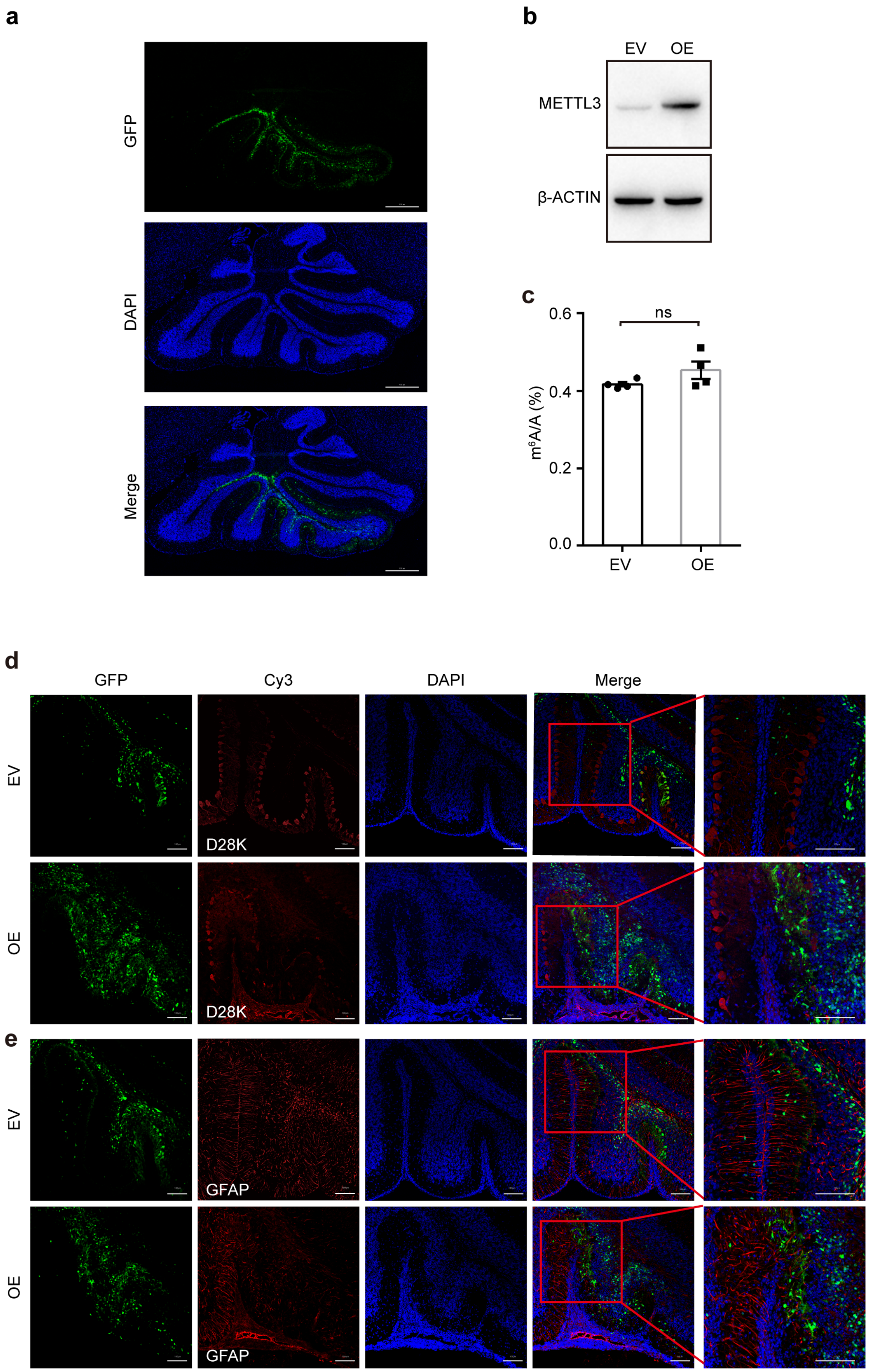

Additional file 1: Figure S5 (Related to Figure 4) Altered morphology in the mouse cerebellum infected with lentivirus for *Mettl3* overexpression. **a** Fluorescence images of cryosections of mouse cerebellum showing the expression of GFP protein in the lobules infected with lentivirus. DAPI staining was performed for nuclei counterstaining. Scale bar represents 500 μm. **b** Western blot results to confirm the overexpression of GFP-METTL3 in Neuro2a cell line. β-ACTIN was used as an internal control. EV, empty vector; OE, *Mettl3* overexpression. **c** UHPLC-MS/MS results showing the change of m^6A levels after local overexpression of *Mettl3* in the mouse cerebellum of P7. n=4 for empty vector or *Mettl3* overexpression, respectively. ns, non-significant. **d, e** Immunofluorescence staining with antibodies against Calbindin-D28K **(d)**, GFAP **(e)** was performed to detect Purkinje cells **(d)** and astrocytes **(e)** upon lentivirus infection. n = 3 biological replicates. GFP fluorescence indicates the area with *Mettl3* overexpression. Sections were counterstained with DAPI to visualize nuclei. Scale bar represents 100 μm.

Ma *et al*., Figure S6, related to Figure 5

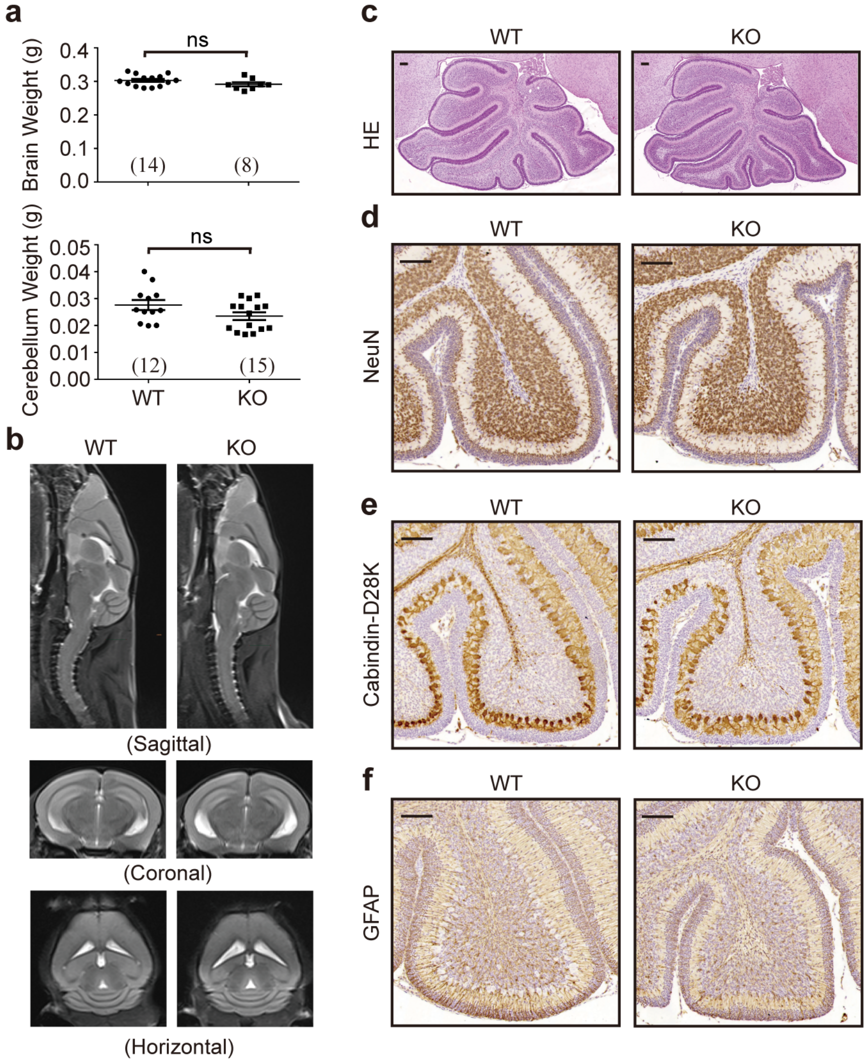

Additional file 1: Figure S6 (Related to Figure 5) Phenotype analysis of the cerebellum in the WT and KO mice under normoxic condition. **a** Brain weight and cerebellar weight of individual wild-type (WT) and *Alkbh5*-knockout (KO) mice of P7. Numbers of biological replicates are included in the parentheses. ns, non-significant. **b** MRI images of individual adult WT and KO mice brains corresponding to the sagittal, coronal and horizontal orientations. Four pairs of mice were examined in total and representative images are shown here. **c** Representative HE staining images of WT and KO mouse cerebellum (P7). n = 5 for WT and KO, respectively. Scale bar, 100 μm. **d-f** Representative images from immunohistochemical analysis with antibodies against NeuN **(d)**, Calbindin-D28K **(e)** and GFAP **(f)** to detect granule cells **(d)**, Purkinje cells **(e)** and astrocytes **(f).** n = 5 for WT and KO, respectively. Scale bar, 100 μm.

Ma *et al.*, Figure S7, related to Figure 5

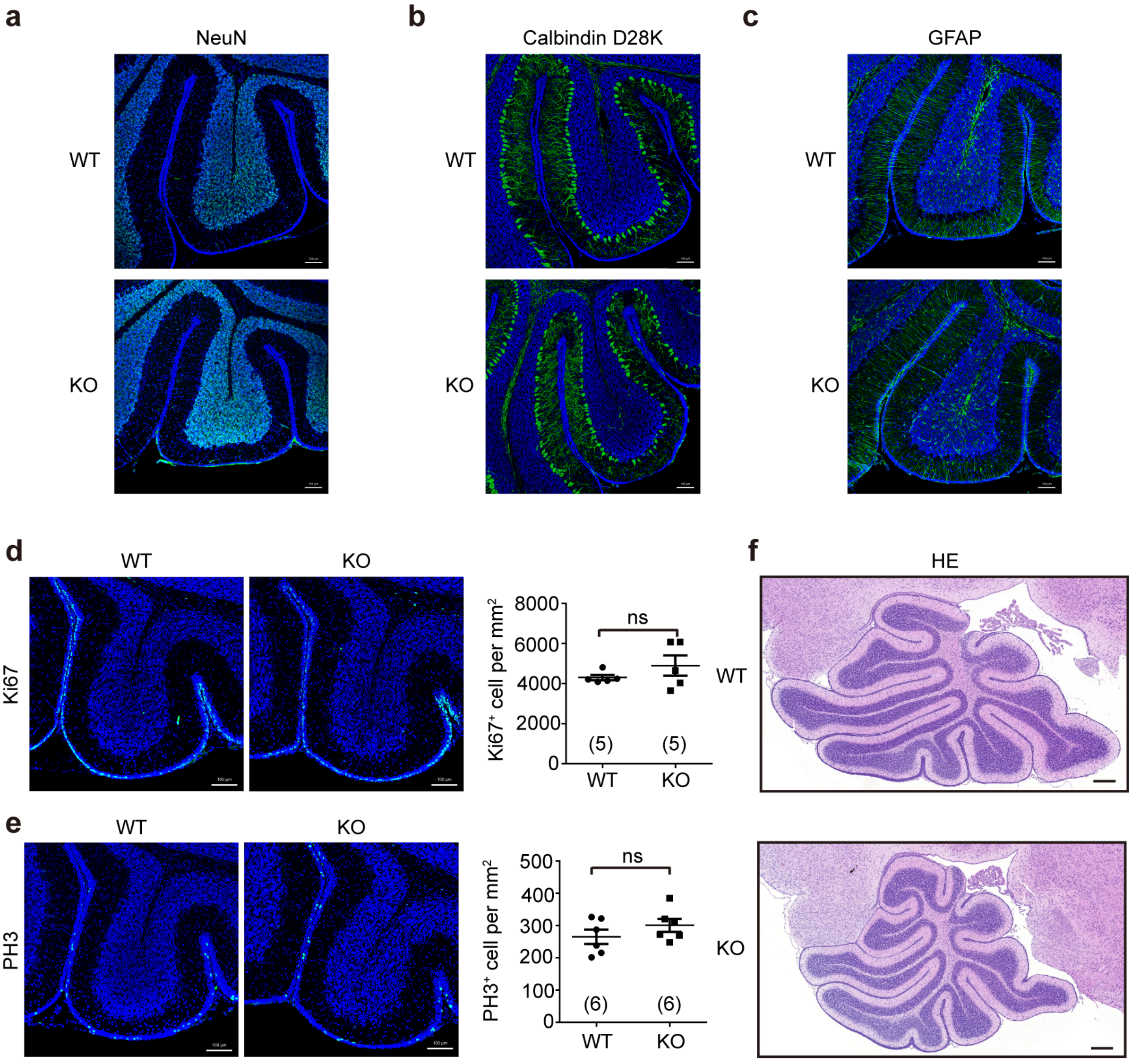

Additional file 1: Figure S7 (Related to Figure 5) Morphology analysis of the cerebellum in WT and KO mice exposed to hypobaric hypoxia and normoxia successively. After being exposed to hypobaric hypoxia, the mice were returned to normoxic condition and raised for another 7 days before dissection. **a-e** Representative images from immunostaining analysis with antibodies against NeuN **(a)**, Calbindin D28K **(b)**, GFAP **(c)**, Ki67 **(d)** and phospho-H3 (PH3) **(e)** in the cerebellum of WT and KO mice. Scale bar, 100 μm. Quantification of immuno-reactive cells in **(d)** and **(e)** is shown in the right panels. Numbers of biological replicates are included in the parentheses. ns, non-significant. **f** Representative HE staining images of WT and KO mouse cerebellum (P7). n = 6 for WT and KO, respectively. Scale bar, 200 μm.

Ma *et al.*, Figure S8, related to Figure 6

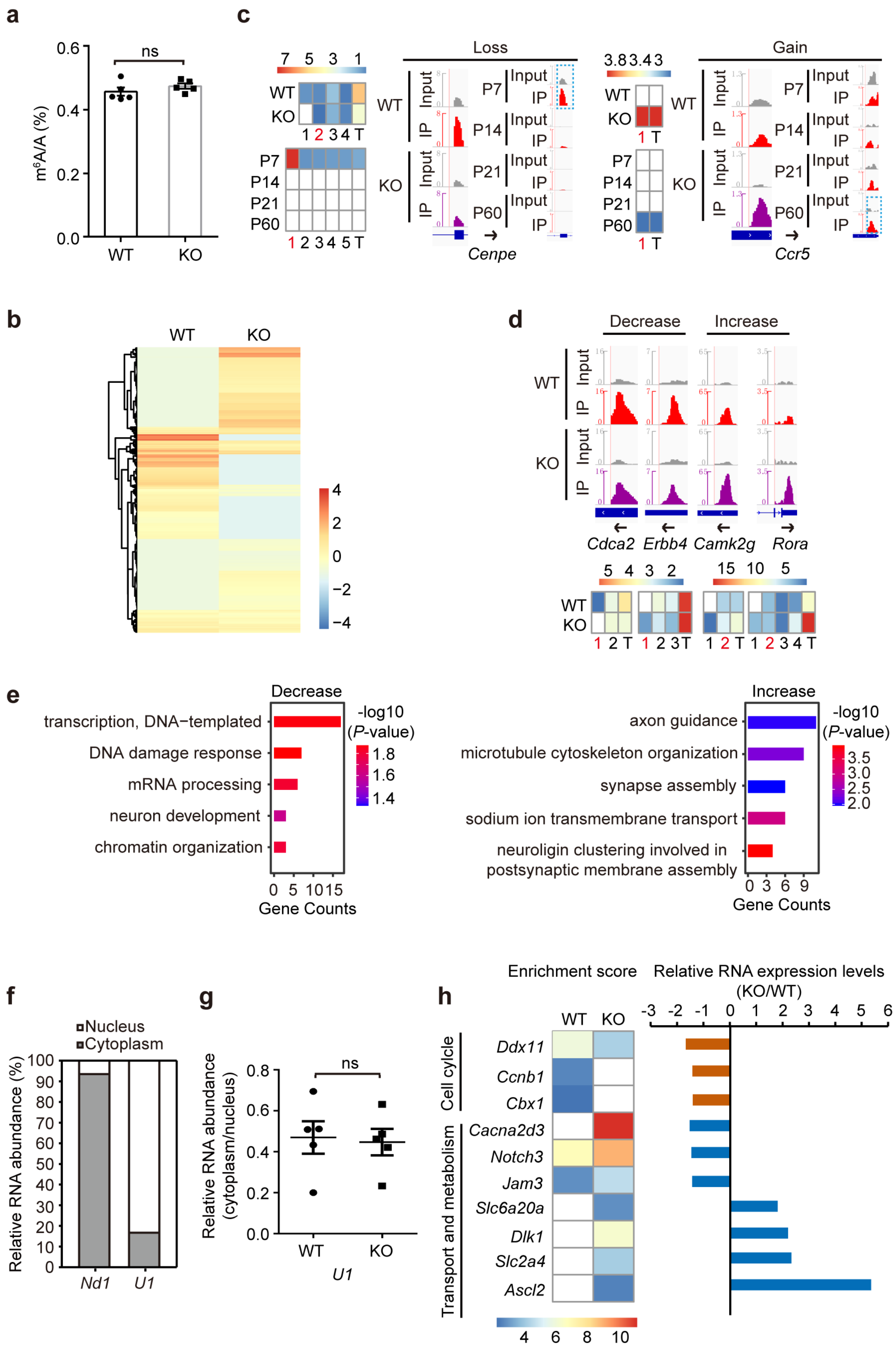

Additional file 1: Figure S8 (Related to Figure 6) Dysregulated RNA methylation resulting from *Alkbh5*-deficiency in mouse cerebellum exposed to hypobaric hypoxia. **a** UHPLC-MS/MS result showing the difference of m^6A methylation levels between WT and KO mouse cerebellar RNAs. n=5 for WT and KO, respectively. ns, non-significant. **b** Heatmap showing the m^6A levels defined by enrichment scores of RNAs with changed methylation in the cerebellum of WT and KO mice. **c** Visualization of the methylation profiles of RNAs with loss- or gain-of-methylation upon *Alkbh5*-deficiency by heatmaps (left panel) and IGV plots (right panel). Heatmaps represent the enrichment scores of each peak and the whole transcript (T). Peaks were numbered according to the transcription direction, and the peaks shown in IGV plots were highlighted in red color. **d** Heatmaps and IGV plots showing examples of RNAs with decreased or increased methylation levels in the cerebellum of KO mice. **e** GO analysis of genes with decreased or increased RNA methylation levels in the cerebellum of the KO mice. **f** Relative RNA abundance of *Nd1* and *U1* in the nuclear and cytoplasmic fractions of mouse cerebellum. *Nd1* and *U1* were used as a marker of cytoplasm and nucleus, respectively. **g** Subcellular localization of *U1* in the cerebellar cells as reflected by its relative RNA abundance in the cytoplasm compared to that in the nucleus. *U1* is shown here as a non-methylated control RNA that is not affected in its subcellular RNA localization. n=10 for WT and KO, respectively. ns, non-significant. **h** Relative expression levels of selected RNAs with altered m^6A levels in the cerebellum of KO mice exposed to hypobaric hypoxia. Column charts in yellow represent the RNAs that are involved in cell cycle control with decreased methylation, and blue columns indicate the RNAs that are involved in transport and metabolism with increased methylation. Heatmaps defined by enrichment scores are included to show the changes in RNA methylation levels.

Additional file 1: Table S1 Data quality and processing information of m^{6}A-seq of poly(A) RNA from wild-type mouse cerebellum (P7, P14, P21 and P60), the cerebellum of wild-type (WT) and *Alkbh5*-knockout (KO) mice exposed to hypobaric hypoxia (P7).

	P7		P14		P21		P60		P7-WT		P7-KO	
	INPUT	IP	INPUT	IP	INPUT	IP	INPUT	IP	INPUT	IP	INPUT	IP
raw data reads	51130796	58498153	56426158	62075940	67033969	63437043	59850210	72249508	40224932	40297706	33424497	44028450
filter low qualitity	49305012	55318385	54364718	58844840	64405326	59442209	57351023	67989711	36016365	36320458	30309449	39516123
length > 70bp	46541745	51656621	51824465	54935713	60821227	55728041	54438571	63445318	34907565	35033205	29378578	38211078
# mapped reads	40452650	46117486	44647785	48276082	52815619	49335915	45880035	55178525	31025285	32059773	26168269	34713750
# unique mapped	38918098	44722529	43117633	46951640	51165644	48033353	44366298	53707803	30041238	31083985	25366417	33740143
% unique mapped	83.6%	86.6%	83.2%	85.5%	84.1%	86.2%	81.5%	84.7%	86.1%	88.7%	86.3%	88.3%

Additional file 1: Table S2 Statistics of m^6A peaks and expressed RNAs in wild-type mouse cerebellum (P7, P14, P21 and P60), the cerebellum of wild-type (WT) and *Alkbh5*-knockout (KO) mice exposed to hypobaric hypoxia (P7). Among all the raw peaks, only the peaks matching FDR<0.05, IP_FPKM >1 and enrichment score > 1.5 were used for subsequent analysis.

	P7	P14	P21	P60	P7-WT	P7-KO
expressed RNAs	19208	18712	18801	18397	19701	20005
m^6A peaks	19459	19599	19550	20755	20722	21940
m^6A RNAs	10449	10266	10419	10783	11215	11852
expressed mRNAs	15050	14753	14513	14624	15041	15157
m^6A mRNAs	9879	9733	9828	10197	10447	10936
methylation ratios	65.6%	66.0%	67.7%	69.7%	69.5%	72.2%
peaks of mRNA	18650	18836	18719	19947	19626	20669
# peaks/transcripts	1.9	1.9	1.9	2.0	1.8	1.9

Additional file 1: Table S3 Numbers of m^6A peaks located in different regions of mRNA transcripts in wild-type mouse cerebellum of P7, P14, P21 and P60.

	P7	P14	P21	P60
5'UTR	725	843	841	999
Start Codon	1986	2254	2172	2798
CDS	5346	5189	5182	5209
Stop Codon	5455	5301	5373	5561
3'UTR	3746	3794	3676	3788
Total	17258	17381	17244	18355

Additional file 1: Table S9 List of antibodies and their applications used in this study.

Antibody	Source	Code	Application
Anti-ALKBH5	Sigma	HPA007196	WB[1], IHC[2]
Anti-β-ACTIN	Santa Cruz	sc-47778	WB
Anti-BrdU	Sigma	B8434	IF[3]
Anti-Calbindin-D28K	Sigma	C9848	IF, IHC
Anti-FTO	Abcam	ab92821	WB, IHC
Anti-GFAP	Sigma	G6171	IF, IHC
Anti-GAPDH	Cell Signaling Technology	2118	WB
Anti-Ki67	Abcam	ab66155	IF
Anti-m^6A	Synaptic Systems	202003	m^6A-IP[4]
Anti-METTL3	Abnova	H00056339-B01P	WB
	Abcam	Ab195352	IHC
Anti-METTL14	Sigma	HPA038002	WB, IHC
Anti-NeuN	Millipore	MAB377	IF, IHC
Anti-PH3	Millipore	06-570	IHC
Anti-WTAP	Santa Cruz	sc-55438	WB
	Proteintech	60188-1-IG	IHC
Anti-Goat IgG (H+L)-HRP conjugated	XI YA Biology	FZ-4211	WB
Anti-Mouse IgG (H+L)-HRP conjugated	XI YA Biology	FZ-4202	WB
Anti-Mouse Ig (peroxidase) Kit	Vector	MP-7402	IHC
Anti-Mouse IgG (H+L), Alex Fluor 594	ThermoFisher Scientific	A11005	IF
Anti-Mouse IgG (H+L), Alex Fluor 488	ThermoFisher Scientific	A11001	IF
Anti-Rabbit IgG (H+L)-HRP conjugated	XI YA Biology	FZ-4201	WB
Anti-Rabbit Ig (peroxidase) Kit	Vector	MP-7401	IHC
Anti-Rabbit IgG (H+L), Alex Fluor 594	ThermoFisher Scientific	A11012	IF
Anti-Rabbit IgG (H+L), Alex Fluor 488	ThermoFisher Scientific	A11008	IF

[1]WB: western-blot

[2]IHC: immunohistochemistry

[3]IF: immunofluorescence

[4]m^6A-IP: m^6A- immunoprecipitation

Additional file 1: Table S10. List of primers for RT-qPCR used in this study.

Gene name	Primer sequence
Gapdh	Forward: 5'-ACAACTTTGGCATTGTGGAA-3'
	Reverse: 5'-GATGCAGGGATGATGTTCTG-3'
Letm1	Forward: 5'-CTCTGCAAGGCTGGTGGTTA-3'
	Reverse: 5'-AGCGATCTTGGTGTCGATCC-3'
Mphosph9	Forward: 5'-GAGTCTGTTGTCAGTTCCCCA-3'
	Reverse: 5'-GTGGGCTCCTTAGCCTTGTG-3'
Nd1	Forward: 5'-AGTTCCCCTACCAATACCACACC-3'
	Reverse: 5'-GGAGTTTGAGGCTCATCCTGATC-3'
Opa1	Forward: 5'-AGACCCCAACTAAGGACACCA-3'
	Reverse: 5'-GTATAGCCACCTCCAACAGCA-3'
U1	Forward: 5'-ATACTTACCTGGCAGGGGAGATACC-3'
	Reverse: 5'-AGCCTCGCCCTGGGAAAA-3'
Wdpcp	Forward: 5'-CCACTGCGTGTCAGTTACCA-3'
	Reverse: 5'-GCCAGGAGAGTCACACCAC-3'
